# Experiences of Domestic Violence and Mental Disorders: A Systematic Review and Meta-Analysis

**DOI:** 10.1371/journal.pone.0051740

**Published:** 2012-12-26

**Authors:** Kylee Trevillion, Siân Oram, Gene Feder, Louise M. Howard

**Affiliations:** 1 Section of Women's Mental Health, Health Service and Population Research Department, Institute of Psychiatry, King's College London, London, United Kingdom; 2 Centre for Academic Primary Care, School of Social and Community Medicine, University of Bristol, Clifton, Bristol, United Kingdom; Maastricht University Medical Centre, The Netherlands

## Abstract

**Background:**

Little is known about the extent to which being a victim of domestic violence is associated with different mental disorders in men and women. We aimed to estimate the prevalence and odds of being a victim of domestic violence by diagnostic category and sex.

**Methods:**

Study design: Systematic review and meta-analysis. Data Sources: Eighteen biomedical and social sciences databases (including MEDLINE, EMBASE, PsycINFO); journal hand searches; scrutiny of references and citation tracking of included articles; expert recommendations, and an update of a systematic review on victimisation and mental disorder. Inclusion criteria: observational and intervention studies reporting prevalence or odds of being a victim of domestic violence in men and women (aged ≥16 years), using validated diagnostic measures of mental disorder. Procedure: Data were extracted and study quality independently appraised by two reviewers. Analysis: Random effects meta-analyses were used to pool estimates of prevalence and odds.

**Results:**

Forty-one studies were included. There is a higher risk of experiencing adult lifetime partner violence among women with depressive disorders (OR 2.77 (95% CI 1.96–3.92), anxiety disorders (OR 4.08 (95% CI 2.39–6.97), and PTSD (OR 7.34 95% CI 4.50–11.98), compared to women without mental disorders. Insufficient data were available to calculate pooled odds for other mental disorders, family violence (i.e. violence perpetrated by a non-partner), or violence experienced by men. Individual studies reported increased odds for women and men for all diagnostic categories, including psychoses, with a higher prevalence reported for women. Few longitudinal studies were found so the direction of causality could not be investigated.

**Conclusions:**

There is a high prevalence and increased likelihood of being a victim of domestic violence in men and women across all diagnostic categories, compared to people without disorders. Longitudinal studies are needed to identify pathways to being a victim of domestic violence to optimise healthcare responses.

## Introduction

Domestic violence is an international public health problem, affecting the lives of hundreds of thousands of people every year. Globally, prevalence estimates of lifetime experiences of physical or sexual partner violence among women range from 15%–71%, with past year estimates ranging from 4% and 54% [1]. No such global estimates exist for men. Research on the prevalence of domestic violence within same-sex relationships is limited; however, evidence from the USA increasingly suggests that the prevalence is similar across same-sex and heterosexual relationships [2].

As a consequence of the substantial physical and psychiatric morbidity associated with domestic violence [3], [4], [5], victims have increased use of health services compared to those not abused [6], [7]. Domestic violence is associated with substantial healthcare costs, with direct medical and mental healthcare costs approximating £1,730 million per annum in the UK and $4.1 billion in the USA, with additional societal costs [8], [9].

Prolonged exposure to threatening life events, including domestic violence, is associated with the onset, duration and recurrence of mental disorders [5], [10], and men and women with mental disorders are at an increased risk of experiencing violence [11]. Recent reviews have suggested that being a victim of domestic violence is common among people with mental disorders [4], [5], [12], [13]. These reviews, however, predominantly focus on depression and PTSD (or report on “mental disorders” without diagnostic characterisation) and have not drawn upon the broader body of research on violence victimisation among people with mental disorders. Furthermore, most reviews do not critically appraise study quality [4], [5], [12] and do not report separately on men who experience domestic violence or on domestic violence perpetrated by family members [4], [12], [13]. This systematic review therefore aimed to estimate:

- The prevalence (lifetime and past year) of being a victim of domestic violence in men and women with mental disorders
- The odds of being a victim of domestic violence in men and women with mental disorders compared with non-mentally disordered controls

## Methods

### Search Strategy

This review followed MOOSE and PRISMA guidelines [14], [15] (see Checklist S1) and the protocol is registered with the PROSPERO database of systematic reviews (http://www.crd.york.ac.uk/prospero); registration number CRD42011001281. A multi-stage search strategy was used, which comprised: (a) an electronic search of 18 bibliographic databases; (b) an update of a recent systematic review on violence experienced by people with mental disorders (i.e. a review which did not focus on domestic violence but may have included studies that collected data on domestic violence) [16]; (c) hand searches of three key journals (i.e. Trauma Violence and Abuse, Journal of Traumatic Stress, and Violence Against Women); (d) screening of references lists of included studies; (e) forwards citation tracking (i.e. identifying studies that had cited the papers included in this review), and (f) expert recommendations. Medical Subject Headings (MeSH) and text words were used to search 18 biomedical and social science electronic databases, from their dates of inception up to 31^st^ March 2011 (see Text S1 for the list of databases used). Terms for domestic violence [17] were adapted from Cochrane protocols and peer-reviewed literature reviews [12], [18] and terms for mental disorders [19] were adapted from NICE guidelines [20] (see Text S2 for Medline, EMBASE and PsycINFO search strategies). When updating the victimisation review [Maniglio [16]], we used the author's original search terms to search databases from September 2007 (the upper limit of the original review) to the 31^st^ March 2011 [16]. No language restrictions were used.

### Selection Criteria

**Figure 1:**
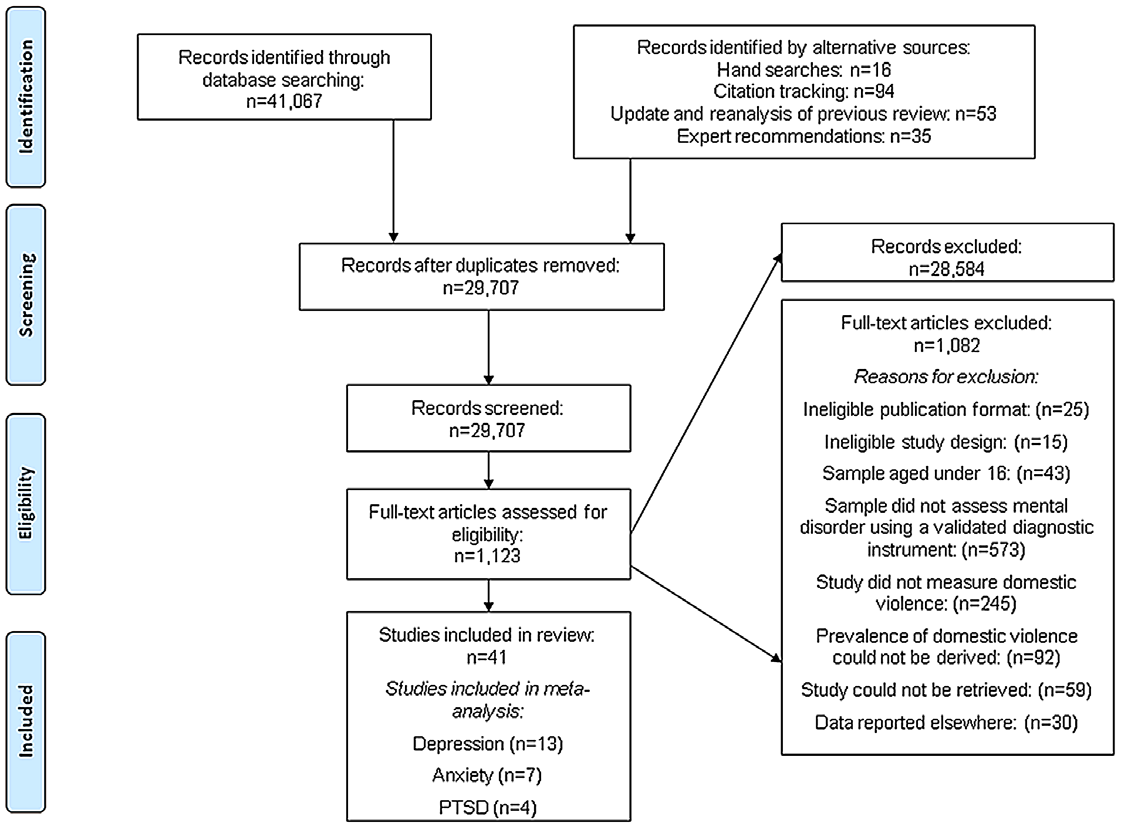
Flow Diagram of Screened and Included Papers.

Studies were eligible for inclusion if they: (a) included men and/or women who were 16 years or older and were diagnosed with a mental disorder using a validated diagnostic instrument (i.e. diagnostic instruments that have been validated against a gold standard measure for diagnosing mental disorder, such as the Schedules for Clinical Assessment in Neuropsychiatry [21]) (see Text S3 for the full definition of mental disorder); (b) presented the results of peer-reviewed research based on experimental studies (e.g. randomised controlled trials, non-randomised controlled trials, parallel group studies), before-and-after studies, interrupted time series studies, cohort studies, case-control studies, or cross-sectional studies; and (c) measured the prevalence or odds of lifetime/past year domestic violence (see Text S3 for the full definition of domestic violence), or reported data from which these statistics could be calculated. In the registered protocol, we stated that studies which used validated screening instruments (i.e. that identify presence of probable mental disorder but do not diagnose mental disorders) would be included in the main text of the review but excluded from meta-analyses. However, due to the large number of screening papers identified (see Figure 1), we decided to include only studies that used validated diagnostic instruments. When we identified multiple eligible papers from the same study only the paper reporting the largest sample size with data of relevance to the objectives of the review was included.

### Data Extraction and Quality Appraisal

The downloaded titles and abstracts were screened against the inclusion criteria by two reviewers (KT and SO). If it was unclear whether a reference met the inclusion criteria, it was taken forward to the next stage of screening. The full texts of potentially eligible studies were assessed by two reviewers (KT and SO). If studies collected data on the prevalence and/or odds of domestic violence but did not report it, authors were contacted for the data. Details of the 1,083 excluded papers and reasons for exclusion are available upon request.

Data from included papers were extracted into a standardised electronic database by two reviewers (KT and SO) and a random sample of 20% was independently cross-checked. Extracted data included details on: the study design; sample characteristics; measures of mental disorder and domestic violence, and the prevalence and odds of lifetime/past year domestic violence. Data were extracted separately for men and women. When reported, details on resource use, impact and severity of violence and chronicity of mental disorders were extracted.

The quality of included studies was independently appraised by two reviewers (KT and SO) using criteria adapted from validated tools [22], [23], [24], [25]. Reviewers compared scores and resolved disagreements before allocating a final appraisal score (see Table S1). Reviewer inter-rater reliability regarding quality scores was high (i.e. for overall quality score: Pearson's r 0.98, ICC 0.95). The quality appraisal checklist includes items assessing study selection and measurement biases (see Table S2). Studies were categorised as high-quality if they scored ≥50% on questions pertaining to selection bias. Quality scoring, particularly for observational research, is contestable [26]; yet we wanted to exclude poor studies that threatened the validity of our findings. The 50% criterion was chosen in order to maximise the number of studies eligible for inclusion in the meta-analysis, while excluding studies in which there was a high risk of selection bias.

### Data Analysis

We calculated prevalence, odds ratios and 95% confidence intervals for domestic violence among men and women by type of mental disorder. If studies measured multiple disorders, odds ratios were calculated separately by type of mental disorder and for each estimate the control group were participants without any mental disorder. Prevalence and odds ratios were also calculated separately by sex and type of violence. Regarding type of violence, we report results for any violence (i.e. physical, sexual and psychological violence) and for physical violence alone. Data on the prevalence and odds of sexual and psychological violence were limited and are given in Table S1. It was not possible to adjust odds ratios for potential confounders (e.g. childhood abuse) due to the lack of data from many of the original studies; unadjusted odds ratios are therefore presented.

We calculated DerSimonian-Laird random effects odds ratio estimates (with corresponding 95% confidence intervals) for lifetime and past year domestic violence among people with mental disorders, compared to people without a mental disorder, if reports were available from three or more high-quality studies [27]. Pooled odds ratios were calculated separately for men and women; studies for which sex-disaggregated data were not available were not eligible for inclusion in the meta-analyses. Disorder-specific summary estimates that included both high- and low-quality papers were calculated in order to assess the impact of excluding low-quality papers. We also examined the influence of individual studies on summary effect estimates by conducting influence analyses, which compute summary estimates omitting one study at a time. Unless stated, neither the inclusion of the low-quality studies nor the omission of individual studies made material differences to odds estimates. We aimed to assess the risk of small study bias with funnel plots in conjunction with Egger's tests [28]. However, due to the small number of eligible studies, statistical tests for funnel plot asymmetry were not appropriate and we were confined to visual inspection of the plots; funnel plots are presented where there were sufficient data-points to allow this (see Figure S1, Figure S2, and Figure S3). Heterogeneity among studies was estimated using the I^2^ statistic (associated 95% confidence intervals were calculated with the STATA *heterogi* command using a non-central χ2 based approach). Due to the small number of studies included in each meta-analysis, it was not possible to use meta-regression to investigate sources of heterogeneity. All analyses were conducted in STATA 11 [29].

## Results

The study selection process is presented in Figure 1. The literature search yielded 29,707 unique references, of which 28,584 were excluded following title and abstract screening. Of the 1,123 references that met, or potentially met, the inclusion criteria, 59 (56 dissertations and three journal articles (in Turkiye'de Psikiyatri, Psikiyatri Psikoloji Psikofarmakoloji Dergis, and Revista de Psiquiatria Clinica) could not be located. Following full text screening, 41 papers were included in the review: 28 were from searches of electronic databases, five from citation tracking, one from hand searching, four from re-examining and updating of an earlier systematic review of victimisation, and three from experts. Six non-English language papers were translated but were not eligible for inclusion in the review.

Key characteristics of the studies are reported in Table 1. Details of design, sample size, definition and measurement of mental disorder and domestic violence are reported in Table S1. Of the 41 included studies, 27 scored ≥50% on quality appraisal criteria for selection bias [30], [31], [32], [33], [34], [35], [36], [37], [38], [39], [40], [41], [42], [43], [44], [45], [46], [47], [48], [49], [50], [51], [52], [53], [54], [55], [56], and 14 scored less than 50% on quality appraisal criteria for selection bias [57], [58], [59], [60], [61], [62], [63], [64], [65], [66], [67], [68], [69], [70]. Unless otherwise stated, results are reported for high-quality studies only.

**Table 1:** Characteristics of included studies (n = 41)*.

	Total (n = 41)	Lifetime domestic violence (n = 26)	Past year domestic violence (n = 18)
Sample:			
Males only	0	0	0
Females only	25	14	14
Males and females	16†	12	4
Diagnoses:			
Schizophrenia & non-affective psychosis	3	2	1
Bipolar affective disorder	2	2	0
Depressive disorders	26	21	12
Dysthymia	5	2	3
Anxiety disorders	15	9	7
PTSD	14	9	7
OCD	2	2	0
Panic disorders	6	2	4
Phobias	3	2	1
Somatisation	1	0	1
Eating disorder	1	0	1
Personality disorder	4	3	1
Common Mental Disorder	5	4	2
Setting:			
Clinical	17	11	7
Non-clinical	24	15	11
Perpetrator:			
Partner only	38‡	24	17
Family only	0	0	0
Partner or family	3	2	1
Type of violence			
Physical violence	20	15	5
Psychological violence	9	5	4
Sexual violence	4	3	1
Physical, sexual, psychological combined	11	5	6
Recency of violence			
Lifetime domestic violence	23		
Past year domestic violence	15	-	-
Lifetime and past year domestic violence	3		
Measurement of domestic violence			
Validated measures	18∞	11	8
Non-validated measures	19◊	11	7
Trauma items from DSM/CIDI criteria	4	4	0
Region:			
North America	17	10	9
Central America	1	1	0
South America	1	1	0
Europe	6	5	2
Africa	3	3	0
Asia	8	6	2
Australasia	5	0	5

* Categories are not mutually exclusive and rows may therefore add to >40.

† Sex-disaggregated data was available for 11 of the 16 studies.

‡ Five papers measured only spousal violence.

∞ Four papers made modifications to validated measures and five did not use all items in the measure.

◊ In 16 studies the authors developed their own measure to assess domestic violence.

### Prevalence and Odds of Domestic Violence by Diagnosis

Results present data for lifetime and past year experiences of any type of partner violence (i.e. physical, sexual and/or psychological violence) across all mental disorders in women and men. Prevalence and odds estimates for all included studies are presented in Table S1; where available, data on specific types of violence (i.e. physical, sexual or psychological violence) and violence by non-intimate family members are also presented in Table S1.

### Depressive Disorders

#### Lifetime Domestic Violence

**Figure 2:**
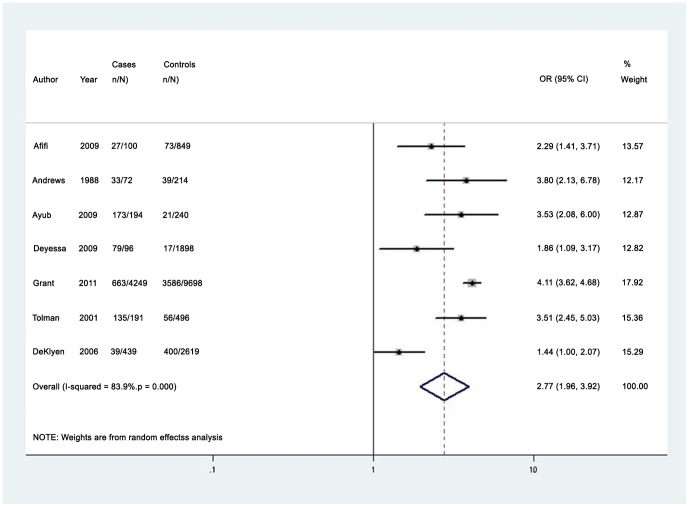
Pooled odds estimates for lifetime intimate partner violence among women with depressive disorders.

Among women, the median prevalence of any lifetime partner violence (7 studies) was 45.8% (IQR 21.3%–76.5%; range 15.6%–89.2%) [30], [36], [38], [46], [50], [52], [55]. The pooled odds ratio for any lifetime partner violence was 2.77 (95% CI 1.96–3.92), with high heterogeneity I^2^ = 83.9% (95% CI 69.0%–92.0%) (See Figure 2) [30], [36], [38], [46], [50], [52], [55]. When excluding one study that used a conservative definition of partner violence, heterogeneity was considerably reduced I^2^ = 61.2% (95% CI 5%–84%), and the revised pooled odds ratio for any lifetime partner violence increased to 3.21 (95% CI 2.49–4.2) [52]. The corresponding funnel plots showed some asymmetry, which may indicate publication bias (Figure S1). Two high-quality studies measured lifetime physical partner violence among men with depressive disorders and reported estimates of 5.3% and 31.3%; both studies reported that men with depressive disorders were more likely to experience domestic violence compared to men with no mental disorders [50], [55].

#### Past Year Domestic Violence

**Figure 3:**
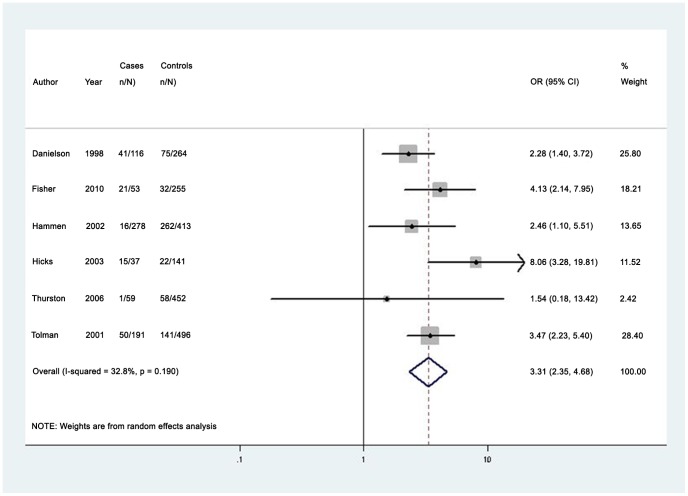
Pooled odds estimates for past year intimate partner violence among women with depressive disorders.

The median prevalence of any past year partner violence (7 studies) was 35.3% (IQR 16.0%–40.1%; range 1.7%–82.5%) among women with depressive disorders [34], [35], [37], [38], [45], [53], [54]. The pooled odds ratio for past year partner violence was 3.31 (95% CI 2.35–4.68); I^2^ = 32.8% (95% CI 0.0%–73.0%) (see Figure 3) [34], [35], [37], [38], [45], [54]. Funnel plots did not indicate asymmetry (see Figure S2). Only one high-quality study reported on any past year domestic violence among men, and identified a prevalence of 80.6% [53].

### Dysthymia

#### Lifetime Domestic Violence

Two high-quality studies reported on the prevalence of any lifetime partner violence among men and women with dysthymia [30], [55]. The largest study (a nationally representative survey of 34,653 non-institutionalised American residents) reported that women with dysthymic disorder had an increased likelihood of experiencing lifetime physical partner violence (OR 5.58 95% CI 4.60–6.76); similar findings were reported among men with dysthymic disorder (OR 4.84 95% CI 2.49–8.79). The study reported lifetime prevalence estimates of 20.0% among women and 3.9% among men with dysthymic disorder [55].

#### Past Year Domestic Violence

A cross-sectional survey of 364 pregnant and postpartum Vietnamese women reported a prevalence of 16.7% for any past year partner violence among six women with dysthymic disorder [54]. No difference in the odds of partner violence were detected among women with dysthymic disorder and women without a mental disorder (OR 1.39 95% CI 0.03–13.00) [54].

### Anxiety Disorders

#### Lifetime Domestic Violence

**Figure 4:**
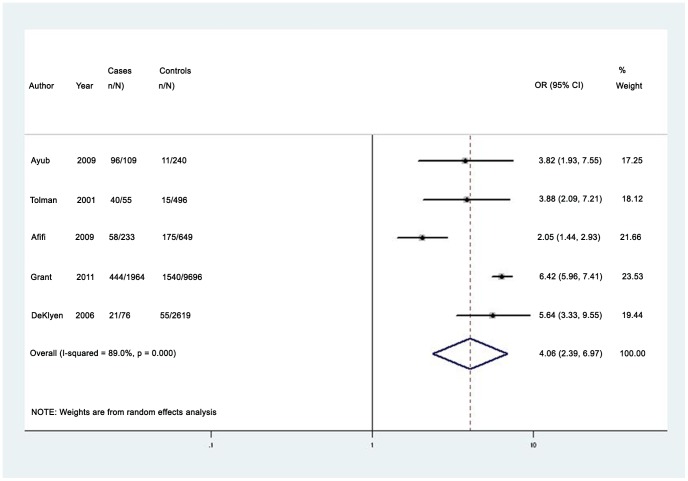
Pooled odds estimates for lifetime intimate partner violence among women with anxiety disorders.

The median prevalence of any lifetime partner violence (5 studies) for women with anxiety disorders was 27.6% (IQR 24.9%–72.7%; range 22.4%–89.9%) [30], [38], [50], [52], [55]. The pooled odds ratio for any lifetime partner violence was 4.08 (95% CI 2.39–6.97), with high heterogeneity (I^2^ = 89.0, 95% CI 77.0%–95.0%) (see Figure 4) [30], [38], [50], [52], [55]. Heterogeneity reduced considerably upon excluding two studies that did not use a validated instrument to measure partner violence (I^2^ = 56.5% (95% CI 0%–88%), and the revised pooled odds ratio for any lifetime partner violence among women with anxiety disorder was 2.92 (95% CI 1.82–4.68) [52], [55]. Only two high-quality studies measured lifetime partner violence among men. These studies found that men with anxiety disorders were significantly more likely to have experienced lifetime physical partner violence than those without a mental disorder, and reported prevalence estimates of 7.4% and 27.0% [50], [55]. Limited data were available on the prevalence and odds of lifetime domestic violence among men and women with phobic [30], [55], [69] or somatoform disorders [69] (see Table S1).

#### Past Year Domestic Violence

**Figure 5:**
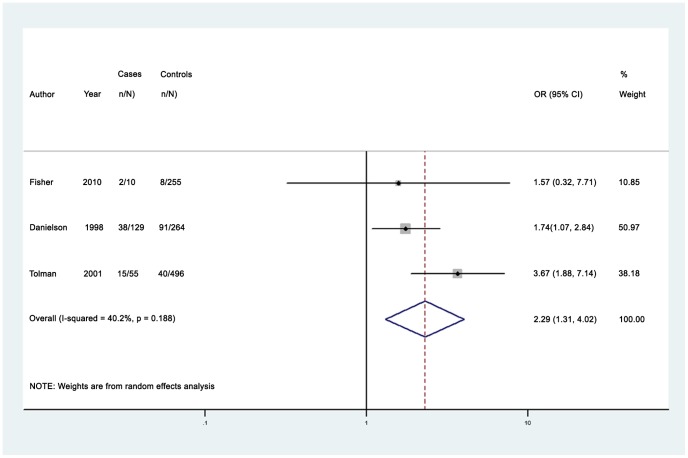
Pooled odds estimates for past year intimate partner violence among women with anxiety disorders.

Among women with anxiety disorders, the median prevalence of any past year partner violence (4 studies) was 28.4% (IQR 25.5%–42.2%, range 20.0%–80.5%) [38], [45], [53], [54]. The pooled odds ratio for any past year partner violence was 2.29 (95% CI 1.31–4.02); (I^2^ = 0.0%, 95% CI 0.0%–90.0%) (see Figure 5) [38], [45], [54]. One high-quality study included men, and reported a prevalence of any past year partner violence of 74.0% among men with anxiety disorders [53].

### Post Traumatic Stress Disorder (PTSD)

#### Lifetime Domestic Violence

**Figure 6:**
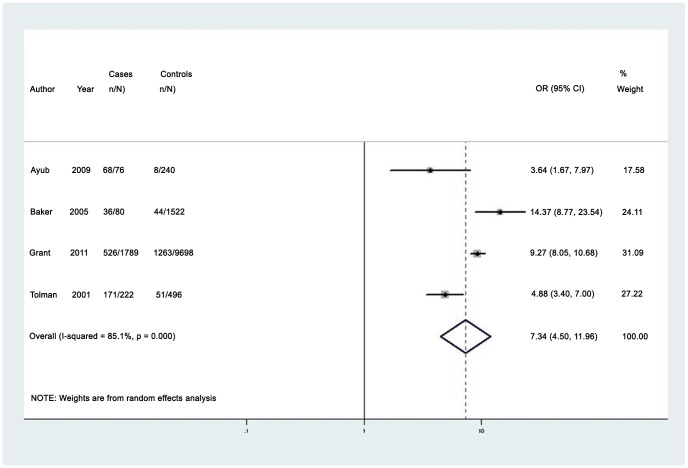
Pooled odds estimates for lifetime intimate partner violence among women with post-traumatic stress disorder.

The median prevalence of any lifetime partner violence (4 studies) among women with PTSD was 61.0% (IQR 41.1%–80.1%; range 29.4%–89.5%) [30], [38], [39], [55]. The pooled odds ratio for any lifetime partner violence was 7.34 (95% CI 4.50–11.98) with high heterogeneity (I^2^ = 85.1%, 95% CI 52.0%–92.0%) (see Figure 6) [30], [38], [39], [55]. Funnel plots did not indicate asymmetry (see Figure S3). One high-quality study included men, and reported an increased likelihood of lifetime physical partner violence among men with PTSD compared to men without a mental disorder (OR 9.66 95% CI 6.49–14.26), with a lifetime prevalence of 7.3% among men with PTSD [55].

#### Past Year Domestic Violence

A survey of female welfare recipients found that 27.0% of women with PTSD had experienced physical partner violence in the past year. The study reported a greater likelihood of past year physical partner violence among women with PTSD compared to women without a mental disorder (OR 3.62; 95% CI 2.32–5.67) [38].

### Obsessive Compulsive Disorder

#### Lifetime Domestic Violence

A cross-sectional survey of 650 women attending primary care clinics in Pakistan reported a prevalence of 93.8% for any lifetime partner violence among women with obsessive compulsive disorder (OCD). The study found that women with OCD had an increased likelihood of experiencing any lifetime partner violence compared to women without a mental disorder (OR 6.43; 95% CI 1.95–33.23) [30].

#### Past Year Domestic Violence

No high-quality studies reported the prevalence or odds of past year domestic violence among men or women with OCD.

### Eating Disorder

#### Lifetime Domestic Violence

No high-quality studies reported the prevalence or odds of lifetime domestic violence among men or women with an eating disorder.

#### Past Year Domestic Violence

One birth cohort study reported that at age 21, 63.6% of 11 women with eating disorders reported past year physical partner violence; women with eating disorders were more likely to report partner violence compared to women without a mental disorder (OR 7.31 95% CI 1.76–35.10) [45].

### Personality Disorder

#### Lifetime Domestic Violence

One study, a national survey of 34,653 non-institutionalised American residents, reported an increased odds of lifetime physical partner violence among both women (OR: 6.06 95% CI 5.35–6.86) and men (OR: 7.04 95% CI 5.30–9.43) with any personality disorder, and lifetime prevalence estimates of 21.4% and 5.4% respectively [55].

#### Past Year Domestic Violence

One birth cohort study reported that at age 21, 100.0% of three women with an antisocial personality disorder reported past year physical partner violence [55].

### Common Mental Disorders (depressive and/or anxiety disorders identified but not disaggregated; CMD)

#### Lifetime Domestic Violence

The median prevalence of any lifetime partner violence among women with CMDs (3 studies) was 48.0% (IQR 35.6%–63.2%, range 23.0%–78.1%); women with CMDs were reported to be more likely to experience any lifetime partner violence compared to those without a mental disorder [42], [43], [44].

#### Past Year Domestic Violence

A national survey of 7,047 UK householders reported an increased odds of any past year partner violence among women (OR: 4.4 95% CI: 3.32–5.82) and men (OR: 3.1 95% CI 2.18–4.39) with CMDs; prevalence estimates were 15.2% and 11.7% respectively [42].

### Schizophrenia and Non-Affective Psychosis

#### Lifetime Domestic Violence

No high-quality studies reported the prevalence or odds of any lifetime partner violence among men or women with schizophrenia and non-affective psychosis. Two lower-quality studies, both conducted with psychiatric samples, reported that the lifetime prevalence of any partner violence ranged from 43.8%–83.3% among women with schizophrenia and non-affective psychosis [59], [64].

#### Past Year Domestic Violence

One birth cohort study reported a prevalence of 43.8% for past year physical partner violence among 16 women with non-affective psychosis [45]. The same study reported that women with non-affective psychosis were more likely to experience past year partner violence compared to women without a mental disorder (OR 3.25; 95% CI 0.97–10.3).

### Bipolar Affective Disorder

#### Lifetime Domestic Violence

One study, a nationally representative survey of 34,563 non-institutionalised American residents, identified an increased odds of lifetime physical partner violence among both women (OR 8.14; 95% CI 6.99–9.47) and men (OR 9.42; 95% CI 6.57–13.50) with bipolar disorder, and lifetime prevalence estimates of 26.7% and 7.1%, respectively [55].

#### Past Year Domestic Violence

No high-quality studies reported the prevalence or odds of past year domestic violence among men or women with bipolar disorder.

### Findings from Longitudinal Studies

Three studies presented longitudinal data on the relationship between mental disorders and domestic violence [36], [51], [53]. A three year cohort study of 286 women found that among 14 women who were depressed and in violent relationship during the study, only one instance of depression predated the violence; rates of depression among the 12 women who had left the violent relationship within one year was no different from that of those who never experienced violence (25% vs. 23%) [36]. Bardone et al, reporting data from the Dunedin birth cohort, found that depression at age 15 did not predict past-year relationship violence at age 21 (OR 1.22 95% CI 0.45–3.03) [51]. Conduct disorder at age 15 was, however, associated with later partner violence (OR 3.14, 95% CI 1.47–6.64). Fergusson et al, reporting data from the Christchurch birth cohort, found that any mental disorder (i.e. depressive, anxiety, conduct, or substance use disorder) at age 14–21 years was associated with past year partner violence at age 24–25 years, and that partner violence and mental disorder at 24–25 years were significantly associated even after adjusting for prior mental disorder and other antecedent and concurrent covariates [53].

### Impact and Resource Use

Limited data were available on the impact of experiences of domestic violence and victims' resource use. Two studies reported increased odds of substance misuse problems (OR of 3.4 and 4.1) among people experiencing domestic violence [41], [47]. One study reported that people experiencing domestic violence had increased odds of suicidal ideation (OR 6.3) [41] and one reported that victims of recent violence experienced greater deprivation (e.g. eviction, homelessness, and food insufficiency) compared to non-victims [38]. Few studies provided details on victims' resource use following domestic violence. One paper reported that women who experienced physical assault (n = 28 women, of which n = 6 reported spousal assault) were significantly more likely to have used emergency services (n = 11, 43%) (p = 0.002) and mental health services (n = 7, 24%) (p<0.001) within the past 12 months [35]. In relation to recency of abuse, another paper found that victims reporting past year violence were twice as likely to have received treatment for mental health problems (1.6%) than victims reporting violence prior to the last twelve months (13.6%) and were twice as likely (26.8% vs.13.6%) to report currently needing treatment [38].

## Discussion

### Key Findings

We found consistent evidence that both men and women with all types of mental disorders report a high prevalence and increased odds of domestic violence compared to people without mental disorder, with women more likely to experience abuse than men. Due to the limited number of high-quality studies it was not possible to calculate pooled odds of partner violence among men or for men or women with disorders other than depression, anxiety or PTSD. Studies on the prevalence and odds of domestic violence by non-intimate family members were also limited. Nonetheless, across a range of diagnoses, studies indicated that men and women with a mental disorder are at an increased likelihood of experiencing domestic violence compared to those without a mental disorder. For example, data from Wave II of the large US National Epidemiologic Survey on Alcohol and Related Conditions suggests that men and women with bipolar affective disorder were more than eight times more likely to report ever having been a victim of partner violence than people with no mental disorder [55].

Although a bi-directional causal relationship between domestic violence and mental disorder seems likely [5], there were insufficient data available from which to draw conclusions about causality. Due to the paucity of longitudinal studies we were only able to make a limited assessment of the temporality of the relationship between mental disorder and domestic violence and of whether recovery from mental disorder is associated with a reduction in risk of domestic violence, or vice versa. It was also not possible, due to insufficient data, to test whether the strength of the association between specific mental disorders and domestic violence varied with severity of violence. We were not able to examine strengths of association between specific mental disorders and recency of domestic violence (i.e. past year vs. lifetime) as odds ratios were calculated from studies with different study populations and measures of violence. This limits our ability to interpret direction of causality here, as recency of mental illness (i.e. past year vs. lifetime) has been shown to affect the strength of association between mental disorder and violence; similarly measurements of lifetime diagnoses may include individuals who may not have experienced a mental disorder during the observation period for acts of violence [71].

### Strengths and Limitations

We used an inclusive search strategy and followed MOOSE and PRISMA reporting guidelines [14], [15]. Our review extends previous research by examining the prevalence and odds of domestic violence across all mental disorders, presenting estimates of the prevalence and odds of domestic violence separately by sex, restricting the scope to studies that used validated diagnostic instruments, and drawing upon the related body of research on mental disorder and victimisation.

All pooled odds ratio estimates indicated that women with mental disorders are at an increased likelihood of experiencing partner violence compared to women without mental disorders. However, in light of the high heterogeneity observed between studies, caution should be exercised when interpreting these figures. Due to a lack of data it was not possible to control for confounding factors when pooling prevalence estimates. When we excluded studies that used conservative definitions of domestic violence or employed non-validated instruments to measure domestic violence, heterogeneity was reduced. However, we do not know the relative contributions of the study setting and measurement of domestic violence to the heterogeneity, and it is likely that study country and known confounding factors (e.g. age, experiences of childhood abuse and substance misuse) may also affect variations in prevalence estimates. Funnel plot asymmetry also indicated the potential for publication bias among studies of depression.

Due to the lack of consistency in the data collected by the primary studies, we were unable to adjust our pooled estimates for potential confounders (e.g. childhood abuse). Furthermore, because of a lack of primary studies, we were unable to: calculate pooled estimates of the odds of domestic violence among men with mental disorders; to assess whether the odds of violence perpetrated by family members was increased among men and women with mental disorder; to analyse whether the prevalence and odds of domestic violence among men and women with mental disorder varied according to sexual preference.

Our meta-analyses were constrained by methodological and conceptual weaknesses in the primary studies. A third of studies scored <50% on quality appraisal criteria relating to selection bias; 23 studies used non-probability sampling, 15 did not provide information on the representativeness of their samples and 14 did not report on the likely impact of non-participation. Although most studies did not score poorly in relation to measurement bias, the measurement of domestic violence varied substantially, with regards to time period (lifetime vs. past year), type of abusive behaviour (physical, sexual, psychological or a combination of behaviours), and instrument. We reported separate estimates of the prevalence and odds of lifetime and past year domestic violence, but recognise that both measures are potentially problematic: recall bias may be present in studies that measure lifetime domestic violence, while participants in studies of past year violence may have had insufficient time to acknowledge or identify their abuse experiences as such [72]. Several papers measured only experiences of physical violence, whereas others included sexual and psychological abuse within their definition of domestic violence. The Conflict Tactics Scale (CTS) was the most commonly used instrument but has been criticised for gender neutrality, measuring acts out of context (not reporting whether acts of violence were in attack or defence) which may lead to differential misclassification bias across sexes. In addition, although the revised CTS partly addresses sexual violence [73], it does not measure other forms of violence [74]. Several papers reported modifying validated instruments without detailing how, if at all, the adapted measures were validated, or reported that they developed their own measures to assess abuse. These factors are likely to reduce both the reliability and comparability of study findings; therefore greater efforts are needed for the development of methodologically robust studies examining the relationship between domestic violence and mental illness [75].

### Implications of Findings

This systematic review provides strong evidence of a high prevalence and increased odds of domestic violence across all mental disorders among both men and women and draws attention to key gaps in the evidence base. The findings of this review highlight the need for healthcare professionals to recognise the increased vulnerability of men and women with mental disorders to domestic violence and to be prepared to identify and address these issues in treatment plans. Current evidence suggests that identification of domestic violence is most effective when professionals are trained to understand the nature of domestic violence and its long term impact on health, to ask about domestic violence safely if abuse is ongoing, and have clear referral and care pathways for identified victims [76], [77]. New guidelines from the World Health Organisation recommend that primary care and mental health services work in partnership with the domestic violence sector to address patients' needs [78]. Further research is needed, however, to investigate which interventions are effective in reducing domestic violence experienced by men and women with mental disorders and how to improve mental health after the abuse has stopped [5].

## Supporting Information

Checklist S1 **PRISMA Checklist.**
(DOC)

Text S1 **Electronic databases searched for systematic review.**
(DOC)

Text S2 **Search Terms for MEDLINE, EMBASE, PsycInfo.**
(DOC)

Text S3 **Definitions of domestic violence and mental disorders.**
(DOC)

Table S1 **Characteristics and reported outcomes of included studies.**
(DOC)

Table S2 **Critical Appraisal Checklist for Included Studies.**
(DOC)

Table S3 **Quality Appraisal Form.**
(DOC)

Figure S1 **Funnel plot: Odds of lifetime domestic violence among women with depressive disorder.**
(TIF)

Figure S2 **Funnel plot: Odds of past year domestic violence among women with depressive disorder.**
(TIF)

Figure S3 **Funnel plot: Odds of lifetime domestic violence among women with post-traumatic stress disorder.**
(TIF)
