# Supplementary material for: Experiences of Domestic Violence and Mental Disorders: A Systematic Review and Meta-Analysis
Source: PLoS One. 2012 Dec 26;7(12):e51740. doi: 10.1371/journal.pone.0051740 (PMC3530507; doi:10.1371/journal.pone.0051740)
Supplement: Text S1 — Electronic databases searched for systematic review. (DOC) [file pone.0051740.s002.doc]

**Biomedical databases**: Academic Search Complete, BNID, CINAHL, Cochrane, EMBASE, HMIC, MEDLINE, Maternity and Infant Care, PsycINFO, Science Direct, Web of Science (including SCI, SSCI, A&HCI, CPCI-S, CPCI-SSH).

**Social sciences databases:** Applied Social Sciences Index and Abstracts, International Bibliography of the Social Sciences, JSTOR, and Sociological Abstracts.

**Theses and dissertations**: DART Europe E Theses Portal, ETHOS, Networked Digital Library of Theses and Dissertations
