# Supplementary material for: Experiences of Domestic Violence and Mental Disorders: A Systematic Review and Meta-Analysis
Source: PLoS One. 2012 Dec 26;7(12):e51740. doi: 10.1371/journal.pone.0051740 (PMC3530507; doi:10.1371/journal.pone.0051740)
Supplement: Text S3 — Definitions of domestic violence and mental disorders. (DOC) [file pone.0051740.s004.doc]

**Definition of Domestic Violence**

“Any incident of threatening behaviour, violence or abuse (psychological, physical, sexual, financial or emotional) between adults who are or have been intimate partners or family members regardless of gender or sexuality”

**Definition of Mental Disorder**

Mental disorder, for the purpose of this review, is defined as including the following diagnoses in accordance with ICD-10 or DSM-IV criteria (or earlier versions of the ICD and DSM classifications): *Schizophrenia, schizotypal and delusional disorders* (Schizophrenia [F20-21, 295.0-6, 295.8-9], other psychoses (schizoaffective disorder [F25, 295.7], paranoid states [297], other nonorganic psychoses [F28-F29, 298], persistent and induced delusional disorders [F22, F24], acute and transient psychotic disorders [F23]); *Mood [affective] disorders* (manic episode [F30, 296.00-.06], bipolar affective disorder [F31, 296.40-.89], depressive disorders [F32-33, 296.20-296.36, 311] (depression in pregnancy and postpartum depression will also be investigated separately), persistent mood [affective] disorder [F34, 300.04, 301.13], other mood [affective] disorder [F38-39]); *Neurotic, stress-related and somatoform disorders* (anxiety disorders [F40-42, 300.01-.03, 300.21-300.29], acute stress reaction [F43, 308.3], post-traumatic stress disorder [F43.1, 309.81], adjustment disorders [F43.2, 309.0-.4, 309.9], dissociative disorders [F44, 300.12-.15, 300.6], somatoform disorders [F45, 300.11, 300.7-300.81, 307.8] other neurotic disorders [F48]); *Eating disorders* [F50.0-F50.9]; and *personality disorders* [F60-61, 301.0-301.9]

N.B. This list excludes primary diagnoses of mental disorder due to psychoactive substance use.  Studies reporting on co-morbid substance use, however, are eligible for inclusion
