# Supplementary material for: Experiences of Domestic Violence and Mental Disorders: A Systematic Review and Meta-Analysis
Source: PLoS One. 2012 Dec 26;7(12):e51740. doi: 10.1371/journal.pone.0051740 (PMC3530507; doi:10.1371/journal.pone.0051740)
Supplement: Table S1 — Characteristics and reported outcomes of included studies. (DOC) [file pone.0051740.s005.doc]

| **Author and year** | **Country** | **Sample size/ gender distribution** | **Method** | **Type of psychiatric disorder** | **Type of domestic violence** | **Prevalence & odds of DV** | **Mean quality appraisal score** |
| --- | --- | --- | --- | --- | --- | --- | --- |
| **Schizophrenia and non-affective psychosis** | | | | | | | |
| Danielson 1998 | New Zealand | 922 male and female sample:  N=461 women  N=480 men  (victimisation not measured in men) | Analysis of data from single wave of cohort study (Dunedin birth cohort, using data collected on psychiatric disorders and violence experienced when respondents aged 21 years)  Domestic violence assessed during interview using Conflict Tactics Scale (CTS)  DSM-III psychiatric disorder assessed during diagnostic interview using DIS | Non-affective psychosis (schizophrenia and schizophreniform disorders)  Frequency of psychosis (women): n=16/461(3.5%)  Frequency of other disorders (women): n=181/461 (39.3%)  Frequency of no disorder (women): n= 264/461 (57.3%) | Past year physical domestic violence by an intimate partner | With non-affective psychosis:  n=7/16 (43.8%)  Without any disorder:  n=51/264 (19.3%)  OR: 3.25 (0.97 – 10.3)  p=0.0192 | Total score:  33/42  Selection bias:  11/14  Measurement bias:  12/14 |
| Friedman 2011 | USA | n= 53 women | Cross sectional survey (women recruited from community mental health centres)  Domestic violence assessed during interviews using author’s questions  DSM-IV psychiatric disorders assessed during diagnostic interview using SCID | Schizophrenia  Frequency of schizophrenia (women): n=6/53 (11.3%)  Frequency of other disorders (women): n=47/53 (88.7%)  Frequency of no disorder (women): n=0/53 (0.0%) | Lifetime violence (physical, sexual, or threatened) by an intimate partner | With schizophrenia:  n=5/6 (83.3%)  Without any disorder:  n/a | Total score:  19/40  Selection bias:  4/14  Measurement bias:  7/14 |
| Wong  2009 | China | 323 women | Cross sectional survey (healthcare survey of ever-married women admitted to nine general hospitals in rural China with suspected suicidal behaviour)  Domestic violence assessed during interview using questions developed by the authors  DSM-IV psychiatric disorders assessed during diagnostic interview using SCID | Non-affective psychosis  Frequency of psychosis (women): n= 24/323 (7.4%) | Lifetime physical domestic violence by a spouse. | With non-affective psychosis:  n=10/24 (41.7%) | Total score:  27/42  Selection bias:  4/14  Measurement bias:  13/14 |
| **Bipolar affective disorder** | | | | | | | |
| Friedman 2011 | USA | n= 53 women | Cross sectional survey (women recruited from community mental health centres)  Domestic violence assessed during interviews using author’s questions  DSM-IV psychiatric disorders assessed during diagnostic interview using SCID | Bipolar I or II disorder  Frequency of bipolar disorder (women):  n=19/53 (35.8%)  Frequency of other disorders (women): n= 34/53 (64.2%)  Frequency of no disorder (women): n=0/53 (0.0%) | Lifetime violence (physical, sexual, or threatened) by an intimate partner. | With bipolar disorder:  n=16/19 (84.2%)  Without any disorder:  n/a | Total score:  19/40  Selection bias:  4/14  Measurement bias:  7/14 |
| Grant  2011 | USA | 34,653 male and female sample:  n= 14,564 men  n= 20,089 women  (Prevalence data are weighted to account for survey design, oversampling and non-response) | Analysis of data from single wave of cohort study ( wave II National Epidemiologic survey on alcohol and related conditions)  Domestic violence assessed during interview using DSM-IV PTSD questions  DSM-IV psychiatric disorders assessed during diagnostic interview using AUDADIS-IV | Bipolar I or II disorder  Frequency of bipolar disorder (men): n=929  Frequency of other disorder (men): n=5,066  Frequency of no disorder (men): n=8,569  Frequency of bipolar disorder (women): n=1,565  Frequency of other disorder (women): n=8,826  Frequency of no disorder (women): n=9,698 | Lifetime physical intimate partner violence | *Men:*  With bipolar disorder: 7.1% (SE 1.14)  Without any disorder:  0.8% (SE 0.11)  OR: 9.42 (6.57-13.50)  p=0.0000    *Women:*  With bipolar disorder: 26.7% (SE 1.36)  Without any disorder:  4.3% (SE 0.26)  OR: 8.14 (6.99-9.47)  p=0.0000 | Total score:  37/42  Selection bias:  13/14  Measurement bias:  13/14 |
| **Depressive disorders** | | | | | | | |
| Afifi  2009 | USA | 2,254 male and female sample:  n= 1116 men  n=1138 women | Cross sectional survey (US National Co-morbidity Survey-Replication)  Domestic violence assessed during interview using adapted Conflict Tactics Scale (CTS)  DSM-IV psychiatric disorder assessed during diagnostic interview using CIDI | Past year mood disorder (combines major depression, dysthymia, mania, bipolar I disorder, and bipolar II disorder)  Frequency of mood disorder (men): n= 64/1116 (5.7%)  Frequency of other disorders (men): n= 135/1116 (12.1%)  Frequency of no disorder (men): n= 917/1116 (82.2%)  Frequency of mood disorder (women): n= 100/1138 (8.8%)  Frequency of other disorders (women): n= 189/1138 (16.6%)  Frequency of no disorder (women): n= 849/1138 (74.6%) | Lifetime physical intimate partner violence | *Men:*  With mood disorder: n=20/64 (31.3%)  Without any disorder: n=152/917 (16.6%)  OR: 2.29 (1.24 – 4.09), p=0.0028  *Women:*  With mood disorder: n=27 /100 (27%)  Without any disorder:  n=118/849 (13.9%)  OR: 2.29 (1.36 – 3.78), p=0.0006 | Total score:  35/42  Selection bias:  12/14  Measurement bias:  11/14 |
| Danielson 1998 | New Zealand | 922 male and female sample:  n=461 women  n=480 men  (victimisation not measured in men | Analysis of data from single wave of cohort study (Dunedin birth cohort, using data collected on psychiatric disorders and violence when respondents aged 21 years)  Domestic violence assessed during interview using Conflict Tactics Scale (CTS)  DSM-III psychiatric disorder assessed during diagnostic interview using DIS | Mood disorder (combines major depressive episode, manic episode and dysthymia) in past 12 months:  Frequency of mood disorder (women): n=116/461 (25.2%)  Frequency of other disorders (women): n= 81/461 (17.5%)  Frequency of no disorder (women): n=264/461 (57.3%) | Past year physical intimate partner violence | With mood disorder: n=41/116(35.3%)  Without any disorder:  n=51/264 (19.3%)  OR: 2.28 (1.36 – 3.82)  p=0.0008 | Total score:  33/42  Selection bias:  11/14  Measurement bias:  12/14 |
| Duran  2009 | USA | 234 women | Cross-sectional survey (outpatient and urgent care clinic of Indian Health Service hospitals in Albuquerque)  Domestic violence assessed during interview using Conflict Tactics Scale (CTS2)  DSM-IV psychiatric disorders assessed during diagnostic interview using CIDI | Mood disorder  Frequency of mood disorder (women): n= 44/234(18.8%)  Frequency of other disorders (women): n= 100/234 (24.7%)  Frequency of no disorder (women): n= 90/234 (38.5%) | Past year physical and sexual intimate partner violence | With mood disorder: n=39/44 (88.6%)  Without any disorder: n=60/90 (66.6%)  OR: 3.9 (1.33 – 13.9)  p=0.0066 | Total score:  27/42  Selection bias:  4/14  Measurement bias:  12/14 |
| Gass  2011 | South Africa | 1,715 male and female sample:  n=641 men  n=1,074 women | Cross-sectional survey (nationally representative community sample of 4,351 South Africans)  Domestic violence assessed during interview using questions adapted from Conflict Tactics Scale (CTS)  DSM-IV psychiatric disorders assessed during diagnostic interview using CIDI | Lifetime mood disorder (combines major depressive disorder, dysthymia)  Frequency of mood disorder (men): n=46/641 (7.2%)  Frequency of mood disorder (women): n= 107/461 (10.0%) | Lifetime physical intimate partner violence | *Men:*  OR: 1.73(0.8-4.0)  Adjusted OR:  2.13 (0.9-5.2)  *Women:*  OR: 1.34(0.9-2.0)  Adjusted OR: 0.81 (0.5-1.4)  *(adjusted odds ratios controlling for significant demographic characteristics and risk factors)* | Total score:  34/42  Selection bias:  11/14  Measurement bias:  12/14 |
| Wong  2009 | China | 323 women | Cross sectional survey (healthcare survey of women admitted to nine general hospitals in rural China with suspected suicidal behaviour)  Domestic violence assessed during interview using questions developed by the authors  DSM-IV psychiatric disorders assessed during diagnostic interview using SCID | Current mood disorder  Frequency of mood disorders (women):  n=86/323 (26.6%) | Lifetime physical spousal violence | With mood disorder: n=18/86 (20.9%) | Total score:  27/42  Selection bias:  4/14  Measurement bias:  13/14 |
| Andrews 1988 | UK | 286 women | Cohort study (longitudinal study data of working class women residing in London borough of Islington)  Authors developed their own question to assess experiences of physical partner violence  Present State Examination (precursor to SCAN) assessed affective disorders | Past year depression  Frequency of depression (women): n=72/286 (25.2%)  Frequency of no disorder (women): n=214/286 (74.8%) | Lifetime physical spousal violence | With depressive disorder:  n=33/72 (45.8%)  Without any disorder: n=39/214 (18.2%)  OR: 3.80 (2.04 - 7.04)  P=0.0000 | Total score:  25/42  Selection bias:  9/14  Measurement bias:  8/14 |
| Ayub  2009 | Pakistan | 650 women | Cross sectional survey (healthcare survey of women attending primary care clinics in Lahore)  Domestic violence assessed during interview using Women Experience with Battering (WEB) and Conflict Tactics Scale (CTS) questionnaires.  ICD-10 psychiatric disorders assessed during diagnostic interview using Mini International Neuropsychiatric Interview | Depression  Frequency of depression (women): n= 194/650 (29.8%)  Frequency of other disorders (women): n= 216/650 (33.2%)  Frequency of no disorder (women): n= 240/650 (36.9%) | Lifetime physical and psychological intimate partner violence | *Any violence:*  With depressive disorder: n=173/194 (89.2%)  Without any disorder: n=168/240 (70%)  OR: 3.53 (2.03 – 6.31)  P=0.0000  *Physical violence*  With depressive disorder: n=89/194 (45.9%)  Without any disorder: n=106/240 (44.2%)  OR: 1.07 (0.72 – 1.60)  P=0.7218  *Psychological violence*  With depressive disorder: n=159/197 (82%)  Without any disorder: n=126/240 (52.5%)  OR: 3.79 (2.40 – 6.02)  P=0.0000 | Total score:  32/42  Selection bias:  8/14  Measurement bias:  12/14 |
| Bardone 1996 | New Zealand | 470 women  (analysis includes 405 women) | Cohort study (Dunedin birth cohort, using data collected on psychiatric disorders aged 15 and violence aged 21 years)  Domestic violence assessed during interview using Conflict Tactics Scale (CTS)  DSM-III psychiatric disorder assessed during diagnostic interview using DIS | Depressive disorder  Frequency of depressive disorder (women): n=27/405 (6.6%)  Frequency of no disorder (women): n= 378/405 (93.3%) | Past year physical intimate partner violence | With depressive disorder: n=8/27 (29.6%)  Without any disorder: n=97/378 (25.7%)  OR: 1.22 (0.45-3.03)  P=0.6494 | Total score:  35/42  Selection bias:  12/14  Measurement bias:  12/14 |
| Cascardi 1995 | USA | 96 women:  n=48 cases (recruited from a treatment study for serious marital conflict)  controls n=48 (recruited maritally discordant non-abused (n=23) and maritally satisfied women from local newspapers (n=25)) | Case control study  (community sample)  Domestic violence assessed during interview using Conflict Tactics Scale (CTS2), Psychological Maltreatment of Women Scale, Spouse-Specific Fear Measure  DSM-III psychiatric disorder assessed during diagnostic interview using SCID | Major depression  Frequency of major depression (women): n=51/96 (53.1%)  Frequency of no disorder (women): data not available | Past year physical and psychological intimate partner violence | With depressive disorder: n=30/51 (58.8%) | Total score:  21/42  Selection bias:  4/14  Measurement bias:  8/14 |
| Cerulli 2011 | USA | 188 women | Cross-sectional (healthcare survey in a paediatric primary care clinic)  Domestic violence assessed during interview using questions adapted from a previous study  DSM-IV psychiatric disorder assessed during diagnostic interview using SCID | Current depression  Frequency of current depression (women): n=106/188 (56.4%)  Frequency of other disorders (women): n= 21/188 (11.2%)  Frequency of no disorder (women): n=61/188 (32.4%) | Lifetime physical, sexual and psychological intimate partner violence | With depressive disorder:  n=31/106 (29.2%)  Without any disorder:  n=8/61 (13.1%)  OR: 2.74 (1.11 – 7.41)  p=0.0177 | Total score:  26/42  Selection bias:  6/14  Measurement bias:  10/14 |
| Chang 2009 | USA | 1,470 male and female sample:  n= 707 men  n= 763 women | Cross-sectional survey (community household sample of Latinos and Asians in the US)  Domestic violence assessed during interview using adapted Conflict Tactics Scale (CTS)  DSM-IV psychiatric disorder assessed during diagnostic interview using CIDI | Current major depression  Prevalence of major depression (men and women): 6.53%  Prevalence of no disorder (men and women): data not available. | Lifetime physical intimate partner violence | *Men:*  Minor violence  OR:2.01 (0.68-5.98)  Severe violence  OR:0.53 (0.06-4.29)  *Women:*  Minor violence  OR:3.72 (1.50-9.26)  Severe violence  OR:5.67 (1.47-21.88) | Total score:  29/42  Selection bias:  10/14  Measurement bias:  10/14 |
| Dennis 2009 | USA | 148 women  n=96 cases  n=52 controls  (both cases and controls recruited from a veteran hospital and a community hospital to obtain mixed sample of veterans and non-veterans) | Case control study (veteran and community healthcare survey)    Domestic violence assessed during interview using the Traumatic Life Events Questionnaire  DSM-IV psychiatric disorder assessed during diagnostic interview using SCID | Major depressive disorder  Frequency of major depressive disorder (women): n= 24/148 (16.2%)  Frequency of other disorders (women): n= 72/148 (48.6%)  Frequency of no disorder (women): n=52/148 (35.1%) | Lifetime physical intimate partner violence | With depressive disorder:  n=12/24 (50%)  Without any disorder:  n=11/52(21.2%)  OR: 3.73 (1.16 – 12.0)  P=0.0109 | Total score:  21/42  Selection bias:  3/14  Measurement bias:  11/14 |
| DeKlyen  2006 | USA | 3077 women | Cohort study (longitudinal cohort of 4,900 births in 20 large US cities between 1998 and 2000)  Domestic violence assessed during interview using questions developed by authors (physical violence by the child’s father assessed)  ICD-10 psychiatric disorders assessed during diagnostic interview using CIDI-Short Form | Depression  Frequency of depression  n= 439/3077 (14.3%)  Frequency of other disorders  n= 76/3077 (2.5%)  Frequency of no disorder  n= 2619/3077 (85.1%) | Lifetime physical partner violence | With depression:  n= 39/439 (8.9%)  Without any disorder:  n= 166/2619 (6.3%)  OR: 1.44 (0.97-2.09)  P=0.0484 | Total score:  34/42  Selection bias:  13/14  Measurement bias:  10/14 |
| Deyessa 2009 | Ethiopia | 1,994 married women | Cross-sectional study (community sample of two Ethiopian districts)  Domestic violence assessed during interview using WHO violence against women questionnaire  ICD-10 psychiatric disorders assessed during diagnostic interview using CIDI | Current depression  Frequency of depression (women): n=96/1,994 (4.8%)  Frequency of no disorder (women): n=1,898/1,994 (95.2%) | Lifetime physical, sexual and psychological intimate partner violence | *Any partner violence:*  With depressive disorder: n=79 /96 (82.3%)  Without any disorder: n=1,356/1,898 (71.4%)  OR: 1.86 (1.08-3.38)  P=0.0210  *Physical violence:*  With depressive disorder: n=69/96 (71.9%)  Without any disorder: n=919/1,898 (48.4%)  OR: 2.72 (1.70 – 4.46)  P=0.0000  *Psychological violence*  With depressive disorder: n=54/96 (56.3%)  Without any disorder: n=499/1,898 (26.3%)  OR: 3.60 (2.33 – 5.60)  P=0.0000  *Sexual violence:*  With depressive disorder: n=60/96 (62.5%)  Without any disorder: n=1,126/1,898 (59.3%)  OR: 1.14 (0.74 – 1.80)  P=0.5365 | Total score:  35/42  Selection bias:  11/14  Measurement bias:  13/14 |
| Fergusson 2005 | New Zealand | 828 male and female sample:  n= 391 men  n= 437 women | Cohort study (birth cohort of 1,265 children born in Christchurch in mid-1977)  Domestic violence assessed during interview using selected items from the Conflict Tactics Scale (CTS2). Non-zero score indicates presence of violence  DSM-IV psychiatric disorders assessed during diagnostic interview using CIDI | Past year major depression  Frequency of major depression (men): n= 31/391 (7.9%)  Frequency of no disorder (men): data not available  Frequency of major depression (women): n=103/437 (23.6%)  Frequency of no disorder (women): data not available | Past year physical, psychological and sexual violence by an intimate partner | *Men:*  With depressive disorder: n=25/31 (80.6%)  *Women:*  With depressive disorder: n=85/103 (82.5%) | Total score:  34/42  Selection bias:  13/14  Measurement bias:  12/14 |
| Fisher 2010 | Vietnam | 364 women | Cross-sectional survey (community sample of women who were >7 months pregnant or 4.8 weeks postpartum and registered with commune health centres in two Vietnamese provinces)  Domestic violence assessed during interview using questions developed by authors. Psychological abuse assessed by inquiring about fear of partner.  DSM-IV psychiatric disorders assessed during diagnostic interview using SCID | Depressive episode  Frequency of depression (women): n= 53/364 (14.6%)  Frequency of other disorders (women): n= 56/364 (15.4%)  Frequency of no disorder (women): n= 255/364 (70.1%) | Past year physical and psychological violence by an intimate partner. | *Any violence*  With depressive disorder:  n=21/53 (39.6%)  Without any disorder:  n=35/255 (13.7%)  OR: 4.13 (2.01 – 8.31)  p=0.0000  *Physical violence*  With depressive disorder:  n=5/53 (9.4%)  Without any disorder:  n=5/255 (2.0%)  OR: 5.21 (1.14 – 23.39)  P=0.0052  *Psychological violence*  With depressive disorder:  n=18/53 (34.0%)  Without any disorder:  n=32/255 (12.5%)  OR: 3.58 (1.70 – 7.39)  p=0.0001 | Total score:  33/42  Selection bias:  12/14  Measurement bias:  11/14 |
| Hammen 2002 | Australia | 816 women (data available on n=691) | Cross sectional survey  Domestic violence assessed during interview using questions from Conflict Tactics Scale (CTS2)  DSM-IV psychiatric disorders assessed during diagnostic interview using SCID | Depression  Frequency of lifetime depression (women): n=278/691 (40.2%)  Frequency of no disorder (women): n=413/691 (59.8%) | Past year physical and psychological domestic violence by an intimate partner | *Lifetime depression*  With depressive disorder:  n= 16/278 (5.8%)  Without any disorder:  n= 10/413 (2.4%)  OR: 2.46 (1.03 – 6.15)  p=0.0239 | Total score:  25/42  Selection bias:  8/14  Measurement bias:  7/14 |
| Friedman 2011 | USA | n= 53 women | Cross sectional survey (women recruited from community mental health centres)  Domestic violence assessed during interviews using author’s questions  DSM-IV psychiatric disorders assessed during diagnostic interview using SCID | Major depression  Frequency of major depression (women): n=28/53 (52.8%)  Frequency of no disorder (women): n/a – psychiatric sample. | Lifetime violence (physical, sexual, or threatened) by an intimate partner | With depressive disorder:  n=15/53 (53.6%) | Total score:  19/40  Selection bias:  4/14  Measurement bias:  7/14 |
| Hicks  2003 | USA | 178 women | Cross sectional survey (community sample, using data from Boston census to obtain representative sample of Chinese citizens)  Domestic violence assessed during interview using questions from Conflict Tactics Scale (CTS)  DSM-IV psychiatric disorders assessed during diagnostic interview using CIDI | Major depression (lifetime)  Frequency of lifetime major depression (women): n=37/178 (20.8%)  Frequency of no disorder (women): n=141/178 (79.2%) | Past year physical domestic violence by an intimate partner | With depressive disorder:  n=15/37 (40.5%)  Without any disorder:  n=11/141 (7.8%)  OR: 8.06 (2.97 – 21.9)  p=0.0000 | Total score:  33/42  Selection bias:  10/14  Measurement bias:  11/14 |
| Lejoyeux 2002 | France | 126 male and female sample:  n= 59 men  n=67 women  (data not disaggregated by gender) | Cross sectional survey (healthcare survey of patients accessing emergency service in district of Paris in 2002)  Domestic violence assessed during interview using adapted questions used in a previous study  DSM-IV psychiatric disorders assessed during diagnostic interview using MINI | Depression  Frequency of depression (men and women): n=14/126 (11.1%)  Frequency of other disorders (men and women): n= 23/126 (18.3%)  Frequency of no disorder (men and women): 89/126 (70.6%) | Lifetime physical, sexual and psychological domestic violence by an intimate partner or family member | *Men and Women*:  With depressive disorder:  n=6/14 (42.9%)  Without any disorder:  n=2/89 (2.2%)  OR: 32.6 (4.53-353.99)  P=0.0000 | Total score:  21/42  Selection bias:  6/14  Measurement bias:  6/14 |
| Grant  2011 | USA | 34,653 male and female sample  n= 14,564 men  n= 20,089 women  (Prevalence data are weighted to account for survey design, oversampling and non-response) | Analysis of data from single wave of cohort study (wave II National Epidemiologic survey on alcohol and related conditions)  Domestic violence assessed during interview using DSM-IV PTSD questions  DSM-IV psychiatric disorders assessed during diagnostic interview using AUDADIS-IV | Major depressive disorder  Frequency of depressive disorder (men):n=1,709  Frequency of other disorder (men): n=4,286  Frequency of no disorder (men): n=8,569  Frequency of depressive disorder (women):n=4,249  Frequency of other disorder (women): n=6,142  Frequency of no disorder (women): n=9,698 | Lifetime physical domestic violence by an intimate partner | *Men:*  With depressive disorder: 5.3% (SE 0.74)  Without any disorder: 0.8% (SE 0.11)  OR: 6.92 (4.99-9.66)  p=0.0000  *Women:*  With depressive disorder: 15.6% (SE 0.66)  Without any disorder: 4.3% (SE 0.26)  OR: 4.44 (3.61-4.69)  p=0.0000 | Total score:  37/42  Selection bias:  13/14  Measurement bias:  13/14 |
| Roberts 1998 | Australia | 335 women  (analysis on available for n=333 women) | Cross sectional survey (healthcare survey of 335 women attending emergency department in Brisbane).  Domestic violence assessed during interview using questions from Composite Abuse Scale (CAS)  DSM-III psychiatric disorders assessed during diagnostic interview using CIDI | Lifetime depression  Frequency of lifetime depression: n=83/333 (24.8%)  Frequency of no disorder (women): data not available | Past year physical, psychological, sexual domestic violence by an intimate partner | With depressive disorder:  n=61/81 (73.5%) | Total score:  30/42  Selection bias:  6/14  Measurement bias:  12/14 |
| Thurston 2006 | Canada | 526 women  (data available for 511 women) | Cross sectional survey (used data from representative community survey of 801 residents in Alberta)  Violence assessed during interview using questions developed by the authors. Domestic violence derived from information on perpetrator of violence.  DSM-IV psychiatric disorders assessed during diagnostic interview using CIDI-SF | Major depressive episode  Frequency of depression (women): n=59/511(11.5%)  Frequency of no disorder (women): n= 452/511 (88.5%) | Past year physical domestic violence by an intimate partner | With depressive disorder:  n=1/59 (1.7%)  Without any disorder:  n=5 /452 (1.1%)  OR: 1.54 (0.32 – 14.1)  P=0.6930 | Total score:  27/42  Selection bias:  8/14  Measurement bias:  8/14 |
| Tolman 2001 | USA | 753 women | Analysis of single wave of cohort study (wave 1 of Women’s Employment Study of female welfare recipients)  Domestic violence assessed during interview using questions from Conflict Tactics Scale (CTS)  DSM-IV psychiatric disorders assessed during diagnostic interview using UM-CIDI. Short form scales were used to assess depression and GAD, PTSD was assessed using the full scale. | Current major depression:  Frequency of current depression (women): n=191/753 (25.4%)  Frequency of other disorders (women): 66/753 (8.8%)  Frequency of no disorder (women): n= 496/753 (65.7%) | Past year and lifetime severe physical domestic violence by an intimate partner | *Lifetime violence*  With depressive disorder:  n=135 /191 (70.7%)  Without any disorder:  n=202/496 (40.7%)  OR: 3.51 (2.42 – 5.12)  P=0.0000  *Past year violence*  With depressive disorder:  n=50/191 (26.2%)  Without any disorder:  n=46/496 (9.3%)  OR: 3.46 (2.17 – 5.53)  P=0.0000 | Total score:  29/42  Selection bias:  9/14  Measurement bias:  12/14 |
| Tuten  2004 | USA | 102 women:  N=26 cases  N=76 controls | Case control study (both cases and controls were recruited from the center for Addictions and Pregnancy service)  Domestic violence assessed using family/partner sections of the Women’s Psychosocial History. Cases are women who reported current partner violence  DSM-III psychiatric disorder assessed during diagnostic interview using SCID-R | Lifetime major depression  Frequency of lifetime major depression (women): n=18/102 (17.6%)  Frequency of no disorder (women): no data available | Current domestic violence (physical, sexual or psychological) by an intimate partner | With depressive disorder:  n=7/18 (38.8%) | Total score:  24/42  Selection bias:  4/14  Measurement bias:  10/14 |
| Vahip  2006 | Turkey | n=100 women | Cross sectional survey (women recruited from psychiatric outpatient clinics)  Method of assessing domestic violence is unclear. Author’s questions used.  DSM-IV psychiatric disorders assessed during diagnostic interview using SCID | Current depression  Frequency of depression (women): n=52/100 (52.0%)  Frequency of no disorder (women): n/a – psychiatric sample | Lifetime physical violence by an intimate partner. | With depressive disorder:  n=34/52 (65.4%) | Total score:  25/40  Selection bias:  5/14  Measurement bias:  8/14 |
| **Anxiety disorders** | | | | | | | |
| Ayub  2009 | Pakistan | 650 women | Cross sectional survey (healthcare survey of women attending primary care clinics in Lahore)  Domestic violence assessed during interview using Women Experience with Battering (WEB) and Conflict Tactics Scale (CTS) questionnaires  ICD-10 psychiatric disorders assessed during diagnostic interview using Mini International Neuropsychiatric Interview | Generalised anxiety disorders (GAD)  Frequency of GAD (women): n=109/650 (16.8%)  Frequency of other disorders (women): n= 301/650 (46.3%)  Frequency of no disorder (women): n= 240/650 (36.9%) | Lifetime physical and psychological intimate partner violence | *Any violence*  With GAD:  n=98/109 (89.9%)  Without any disorder: n=168/240 (70%)  OR: 3.81 (1.89 – 8.35)  p=0.0001  *Physical violence*  With GAD:  n=45/109 (41.3%)  Without any disorder: n=106/240 (44.2%)  OR: 0.89 (0.55 – 1.44)  P=0.6145  *Psychological violence*  With GAD:  n=95/109 (87.2%)  Without any disorder: n=126/240 (52.5%)  OR: 6.14 (3.24 – 12.27)  P=0.0000 | Total score:  32/42  Selection bias:  8/14  Measurement bias:  12/14 |
| Cascardi 1995 | USA | 96 women:  n=48 cases  controls n=48 | Case control study (cases recruited from a treatment study on serious marital conflict and controls recruited from local newspapers, participants were maritally discordant non-abused (n=23) and martially satisfied women (n=25))  Domestic violence assessed during interview using Conflict Tactics Scale (CTS2), Psychological Maltreatment of Women Scale, Spouse-Specific Fear Measure  DSM-III psychiatric disorder assessed during diagnostic interview using SCID | Generalised Anxiety Disorder (GAD)  Frequency of GAD(women): n= 12/96 (12.5%)  Frequency of no disorder (women): data not available | Past year physical, psychological domestic violence by an intimate partner | With GAD:  n=5 /12 (41.7%) | Total score:  21/42  Selection bias:  4/14  Measurement bias:  8/14 |
| Fisher 2010 | Vietnam | 364 women | Cross-sectional survey (community sample of women who were >7 months pregnant or 4.8 weeks postpartum and registered with commune health centres in two Vietnamese provinces)  Domestic violence assessed during interview using questions developed by authors. Psychological abuse assessed by inquiring about fear of partner  DSM-IV psychiatric disorders assessed during diagnostic interview using SCID | Generalised Anxiety Disorder (GAD)  Frequency of GAD (women): n=10/364 (2.7%)  Frequency of other disorders (women): n= 99/364 (27.2%)  Frequency of no disorder (women): n= 255/364 (70.1%) | Past year physical and psychological violence by an intimate partner. | *Any violence*  With GAD:  n=2/10 (20.0%)  Without any disorder:  n=35/255 (13.7%)  OR: 1.57 (0.16 – 8.32)  P=0.5744  *Physical violence*  With GAD:  n=0/10 (0.0%)  Without any disorder:  n=5/255 (2.0%)  OR: n/a  *Psychological violence*  With GAD:  n=2/10 (20.0%)  Without any disorder:  n=32/255 (12.5%)  OR: 1.74 (0.17 – 9.26)  P=0.4895 | Total score:  33/42  Selection bias:  12/14  Measurement bias:  11/14 |
| Grant  2011 | USA | 34,653 male and female sample:  n= 14,564 men  n= 20,089 women  (Prevalence data are weighted to account for survey design, oversampling and non-response) | Analysis of data from single wave of cohort study ( wave II National Epidemiologic survey on alcohol and related conditions)  Domestic violence assessed during interview using DSM-IV PTSD questions  DSM-IV psychiatric disorders assessed during diagnostic interview using AUDADIS-IV | Generalised Anxiety Disorder (GAD)  Frequency of GAD (men): n=746  Frequency of other disorder (men): n=5,249  Frequency of no disorder (men): n=8,569  Frequency of GAD (women): n=1,984  Frequency of other disorder (women): n=8,407  Frequency of no disorder (women): n=9,698 | Lifetime physical domestic violence by an intimate partner | *Men*  With anxiety disorder: 7.4% (SE 1.11)  Without any disorder:  0.8% (SE 0.11)  OR: 9.81 (6.69-14.30)  p=0.0000  *Women*  With anxiety disorder: 22.4% (SE 1.13)  Without any disorder:  4.3% (SE 0.26)  OR: 6.42 (5.54-7.43)  p=0.0000 | Total score:  37/42  Selection bias:  13/14  Measurement bias:  13/14 |
| Roberts 1998 | Australia | 335 women  (data on DV and MD available for 333) | Cross sectional survey (healthcare survey of 335 women attending emergency department in Brisbane)  Domestic violence assessed during interview using questions from Composite Abuse Scale (CAS)  DSM-III psychiatric disorders assessed during diagnostic interview using CIDI | Lifetime Generalised Anxiety Disorders (GAD)  Frequency of GAD (women):  n=32/333 (9.6%)  Frequency of no disorder (women): data not available | Past year physical, psychological, sexual domestic violence by an intimate partner | With anxiety disorder:  n=26/32 (81.3%) | Total score:  30/42  Selection bias:  6/14  Measurement bias:  12/14 |
| Tolman 2001 | USA | 753 women | Analysis of single wave of cohort study (wave 1 of Women’s Employment Study of female welfare recipients)  Domestic violence assessed during interview using questions from Conflict Tactics Scale (CTS)  DSM-IV psychiatric disorders assessed during diagnostic interview using UM-CIDI. Short form scales were used to assess depression and GAD, PTSD was assessed using the full scale. | Current Generalised Anxiety Disorder (GAD)  Frequency of GAD (women): n= 55/753 (7.3%)  Frequency of other disorders (women): 202/753 (26.8%)  Frequency of no disorders (women): n=496/753 (65.9%) | Past year and lifetime severe physical domestic violence by an intimate partner | *Lifetime violence*  With GAD:  n=40 /55 (72.7%)  Without any disorder:  n=202/496 (40.7%)  OR: 3.88 (2.03 – 7.76)  p=0.0000  *Past year violence*  With GAD:  n=15/55 (27.3%)  Without any disorder:  n=46/496 (9.3%)  OR: 3.66 (1.74 – 7.40)  p=0.0001 | Total score:  29/42  Selection bias:  9/14  Measurement bias:  12/14 |
| Afifi  2009 | USA | 2,254 male and female sample:  n= 1116 men  n=1138 women | Cross sectional survey (US National Co-morbidity Survey-Replication)  Domestic violence assessed during interview using adapted Conflict Tactics Scale (CTS)  DSM-IV psychiatric disorder assessed during diagnostic interview using CIDI | Past year anxiety disorder  Frequency of anxiety disorder (men): n=122/1,116 (10.9%)  Frequency of other disorders (men): 77 /1,116 (6.9%)  Frequency of no disorder (men): 917/1,116 (82.2%)  Frequency of anxiety disorder (women): n=233/1,138 (20.5%)  Frequency of other disorders (women): n= 56/1,138 (4.9%)  Frequency of no disorder (women): n= 849/1,138 (74.6%) | Lifetime physical domestic violence by an intimate partner. | *Men*  With anxiety disorder:  n=33/122 (27%)  Without any disorder: n=152/917 (16.6%)  OR: 1.87 (1.17 – 2.93)  p=0.0045  *Women:*  With anxiety disorder:  n=58/233 (24.9%)  Without any disorder:  n=118/849 (13.9%)  OR: 2.05 (1.41 – 2.96)  p=0.0001 | Total score:  35/42  Selection bias:  12/14  Measurement bias:  11/14 |
| Danielson 1998 | New Zealand | 922 male and female sample:  N=461 women  N=480 men  (victimisation not measured in men) | Analysis of data from single wave of cohort study (Dunedin birth cohort, using data collected on psychiatric disorders and violence when respondents aged 21 years)  Domestic violence assessed during interview using Conflict Tactics Scale (CTS)  DSM-III psychiatric disorder assessed during diagnostic interview using DIS | Anxiety disorder  Frequency of anxiety disorder (women): n=129/461 (27.9%)  Frequency of other disorders (women): n= 68/461(14.8%)  Frequency of no disorder (women): n= 264/461 (57.3%) | Past year physical domestic violence by an intimate partner | With anxiety disorder:  n=38/129(29.4%)  Without any disorder:  n=51/264 (19.3%)  OR: 1.74 (1.04 – 2.91)  p=0.0241 | Total score:  33/42  Selection bias:  11/14  Measurement bias:  12/14 |
| DeKlyen  2006 | USA | 3077 women | Cohort study (longitudinal cohort of 4,900 births in 20 large US cities between 1998 and 2000)  Domestic violence assessed during interview using questions developed by authors (physical violence by the child’s father assessed)  DSM-IV psychiatric disorders assessed during diagnostic interview using CIDI-Short Form | Anxiety disorder  Frequency of anxiety disorder:  n= 76/3077 (2.5%)  Frequency of other disorders:  n= 382/3077 (12.4%)  Frequency of no disorder:  n= 2619/3077 (85.1%) | Lifetime physical partner violence | With anxiety disorder:  n= 21/76 (27.6%)  Without any disorder:  n= 166/2619 (6.3%)  OR: 5.64 (3.16-9.74)  P<0.0001 | Total score:  34/42  Selection bias:  13/14  Measurement bias:  10/14 |
| Duran 2009 | USA | 234 women | Cross-sectional survey (outpatient and urgent care clinic of Indian Health Service hospital in Albuquerque)  Domestic violence assessed during interview using Conflict Tactics Scale (CTS2)  DSM-IV psychiatric disorders assessed during diagnostic interview using CIDI | Anxiety disorder  Frequency of anxiety disorder (women): n=106/234 (45.3%)  Frequency of other disorder (women): n=38/234 (16.2%)  Frequency of no disorder (women): n=90/234 (38.5%) | Past year physical and sexual domestic violence by an intimate partner | With anxiety disorder:  n=91/106 (85.8%)  Without any disorder:  n=60/90 (66.7%)  OR: 3.03 (1.42 – 6.58)  p=0.0015 | Total score:  27/42  Selection bias:  4/14  Measurement bias:  12/14 |
| Fergusson 2005 | New Zealand | 828 male and female sample:  n= 391 men  n= 437 women | Cohort study (birth cohort of 1,265 children born in Christchurch in mid-1977)  Domestic violence assessed during interview using selected items from the Conflict Tactics Scale (CTS2). Non-zero score indicates presence of violence  DSM-IV psychiatric disorders assessed during diagnostic interview using CIDI | Past year anxiety disorder  Frequency of anxiety disorder (men): n=27/391 (6.9%)  Frequency of no disorder (men): data not available  Frequency of anxiety disorder in women: n=82/437 (18.8%)  Frequency of no disorder (women): data not available | Past year violence (physical, psychological and sexual) violence by an intimate partner | *Men*  With anxiety disorder:  n=20/27 (74.0%)  *Women*  With anxiety disorder:  n=66/82 (80.5%) | Total score:  34/42  Selection bias:  13/14  Measurement bias:  12/14 |
| Gass  2011 | South Africa | 1,715 male and female sample:  n=641 men  n=1,074 women | Cross-sectional survey (nationally representative community sample of 4,351 South Africans)  Domestic violence assessed during interview using questions adapted from Conflict Tactics Scale (CTS)  DSM-IV psychiatric disorders assessed during diagnostic interview using CIDI | Lifetime anxiety disorder;  Frequency of anxiety disorder (men): n= 59/641 (9.2%)  Frequency of no disorder (men): data not available  Frequency of anxiety disorder (women): n=170/1,074 (15.8%)  Frequency of no disorder (women): data not available | Lifetime physical domestic violence by an intimate partner | *Men*  Anxiety disorder OR:  0.89(0.4-1.9)  Adjusted OR:  0.54(0.2-1.9)  *Women*  Anxiety disorder OR:  2.19(1.4-3.4)  Adjusted OR:  1.44(0.8-2.6)  *(adjusted odds ratios controlling for significant demographic characteristics and risk factors)* | Total score:  34/42  Selection bias:  11/14  Measurement bias:  12/14 |
| Lejoyeux 2002 | France | 126 male and female sample:  n= 59 men  n=67 women  (data not disaggregated by gender) | Cross sectional survey (healthcare survey of patients accessing emergency service in district of Paris in 2002)  Domestic violence assessed during interview using adapted questions used in a previous study  DSM-IV psychiatric disorders assessed during diagnostic interview using MINI | Anxiety disorder  Frequency of anxiety disorder (men and women) n= 15/126 (11.9%)  Frequency of other disorders (men and women): n=22/126 (17.5%)  Frequency of no disorder (men and women): n=89/126 (70.6%) | Lifetime physical, sexual and psychological domestic violence by an intimate partner or family member | *Men and Women:*  With anxiety disorder:  n=13/15 (86.7%)  Without any disorder:  n=2/89 (2.2%)  OR: 282.8 (29.3 – 3464.45)  p=0.0000 | Total score:  21/42  Selection bias:  6/14  Measurement bias:  6/14 |
| Tuten  2004 | USA | 102 women:  N=26 cases  N=76 controls  (both cases and controls were recruited from the Center for Addictions and Pregnancy service) | Case control study (healthcare setting, residential treatment for addiction and pregnancy)  Domestic violence assessed using family/partner sections of the Women’s Psychosocial History.  DSM-III psychiatric disorder assessed during diagnostic interview using SCID-R | Lifetime anxiety disorders    Frequency of lifetime anxiety disorder (women): n=18/102 (17.6%)  Frequency of no disorder (women): data not available | Current physical, sexual and psychological domestic violence by an intimate partner | *Lifetime anxiety*  With anxiety disorder:  n=5/18 (27.8%) | Total score:  24/42  Selection bias:  4/14  Measurement bias:  10/14 |
| Vahip  2006 | Turkey | n=100 women  . | Cross sectional survey (women recruited from psychiatric outpatient clinics)  Method of assessing domestic violence is unclear. Author’s own questions used.  DSM-IV psychiatric disorders assessed during diagnostic interview using SCID | Anxiety disorder  Frequency of anxiety disorder (women): n= 7/100 (7.0%)  Frequency of no disorder (women): n/a – psychiatric sample | Lifetime physical violence by an intimate partner. | With anxiety disorder:  n=3/7(42.9%) | Total score:  25/40  Selection bias:  5/14  Measurement bias:  8/14 |
| Ayub  2009 | Pakistan | 650 women | Cross sectional survey (healthcare survey of women attending primary care clinics in Lahore)  Domestic violence assessed during interview using Women Experience with Battering (WEB) and Conflict Tactics Scale (CTS) questionnaires  ICD-10 psychiatric disorders assessed during diagnostic interview using Mini International Neuropsychiatric Interview | Panic disorder  Frequency of panic disorder (women): n= 74/650 (11.4%)  Frequency of other disorders (women): n= 336/650 (51.7%)  Frequency of no disorder (women): n= 240/650 (36.9%) | Lifetime physical and psychological intimate partner violence | *Any violence*  With panic disorder:  n=62/74 (83.8%)  Without any disorder: n=168/240 (70%)  OR: 2.21 (1.10 – 4.78)  p=0.0192  *Physical violence*  With panic disorder:  n=43/74 (58.1%)  Without any disorder: n=106/240 (44.2%)  OR: 1.75 (1.00 – 3.08)  P=0.0358  *Psychological violence*  With panic disorder:  n=50/74 (67.6%)  Without any disorder: n=126/240 (52.5%)  OR: 1.88 (1.06 – 3.42)  p=0.0224 | Total score:  32/42  Selection bias:  8/14  Measurement bias:  12/14 |
| Cascardi 1995 | USA | 96 women:  n=48 cases (recruited from a treatment study for serious marital conflict)  controls n=48 (recruited maritally discordant non-abused (n=23) and martially satisfied women from local newspapers (n=25)) | Case control study (community sample, cases recruited from a treatment study for serious marital conflict, controls (maritally discordant non-abused and martially satisfied women) recruited from local newspapers)  Domestic violence assessed during interview using Conflict Tactics Scale (CTS2), Psychological Maltreatment of Women Scale, Spouse-Specific Fear Measure  DSM-III psychiatric disorder assessed during diagnostic interview using SCID | Panic disorder  Frequency of panic disorder (women): n= 12/96 (12.5%)  Frequency of no disorder (women): data not available | Past year physical, psychological domestic violence by an intimate partner | With panic disorder: n= 6/12 (50.0%) | Total score:  21/42  Selection bias:  4/14  Measurement bias:  8/14 |
| Cerulli 2011 | USA | 188 women | Cross-sectional (healthcare survey in a paediatric primary care clinic)  Domestic violence assessed during interview using questions adapted from a previous study    DSM-IV psychiatric disorder assessed during diagnostic interview using SCID | Panic disorder  Frequency of current panic disorder (women): n= 7/188 (3.7%)  Frequency of other disorder (women): n= 120/188 (63.8%)  Frequency of no disorder (women): n= 61/188 (32.4%) | Lifetime physical, sexual and psychological partner violence | With panic disorder:  n=4/7 (57.1%)  Without any disorder:  n=8/61 (13.1%)  OR: 8.83 (1.19 – 68.61)  p=0.0038 | Total score:  26/42  Selection bias:  6/14  Measurement bias:  10/14 |
| Fisher 2010 | Vietnam | 364 women | Cross-sectional survey (community sample of women who were >7 months pregnant or 4.8 weeks postpartum and registered with commune health centres in two Vietnamese provinces)  Domestic violence assessed during interview using questions developed by authors. Psychological abuse assessed by inquiring about fear of partner  DSM-IV psychiatric disorders assessed during diagnostic interview using SCID | Panic disorder  Frequency of panic disorder (women): n= 27/364 (7.4%)  Frequency of other disorders (women): n= 82/364 (22.5%)  Frequency of no disorders (women): n= 255/364 70.1%) | Past year physical and psychological violence by an intimate partner. | *Any violence*  With panic disorder:  n=6/27 (22.2%)  Without any disorder:  n=35/255 (13.7%)  OR: 1.80 (0.55 – 5.02)  p=0.2336  *Physical violence*  With panic disorder:  n=1/27 (3.7%)  Without any disorder:  n=5/255 (2.0%)  OR: 1.92 (0.04 – 18.11)  p=0.5506  *Psychological violence*  With panic disorder:  n=6/27 (22.2%)  Without any disorder:  n=32/255 (12.5%)    OR: 1.99 (0.61 – 5.60)  p=0.1616 | Total score:  33/42  Selection bias:  12/14  Measurement bias:  11/14 |
| Grant  2011 | USA | 34,653 male and female sample:  n= 14,564 men  n= 20,089 women    (Prevalence data are weighted to account for survey design, oversampling and non-response) | Analysis of data from single wave of cohort study (wave II National Epidemiologic survey on alcohol and related conditions)  Domestic violence assessed during interview using DSM-IV PTSD questions  DSM-IV psychiatric disorders assessed during diagnostic interview using AUDADIS-IV | Panic disorder  Frequency of panic disorder (men): n=708  Frequency of other disorder (men): n=5,287  Frequency of no disorder (men): n=8,569  Frequency of panic disorder (women): n=1,919  Frequency of other disorder (women): n=8,472  Frequency of no disorder (women): n=9,698 | Lifetime physical domestic violence by an intimate partner | *Men*  With panic disorder: 7.2% (SE 1.18)  Without any disorder:  0.8% (SE 0.11)  OR: 9.56 (6.46-14.06)  p=0.0000  *Women*  With panic disorder: 22.9% (SE 1.14)  Without any disorder:  4.3% (SE 0.26)  OR: 6.60 (5.70-7.65)  p=0.0000 | Total score:  37/42  Selection bias:  13/14  Measurement bias:  13/14 |
| Ayub  2009 | Pakistan | 650 women | Cross sectional survey (healthcare survey of women attending primary care clinics in Lahore)  Domestic violence assessed during interview using Women Experience with Battering (WEB) and Conflict Tactics Scale (CTS) questionnaires  ICD-10 psychiatric disorders assessed during diagnostic interview using Mini International Neuropsychiatric Interview | Agoraphobia  Frequency of agoraphobic disorder (women): n= 70/650 (10.8%)  Frequency of other disorders (women): n= 340/650 (52.3%)  Frequency of no disorder (women): n= 240/650 (36.9%) | Lifetime physical and psychological intimate partner violence | *Any violence*  With agoraphobia:  n=56/70 (80%)  Without any disorder: n=168/240 (70%)  OR: 1.71 (0.87 – 3.55)  p=0.1001  *Physical violence*  With agoraphobia:  n=46/70 (65.7%)  Without any disorder: n=106/240 (44.2%)  OR: 2.42 (1.35 – 4.42)  p=0.0015  *Psychological violence*  With agoraphobia:  n=44/70 (62.9%)  Without any disorder: n=126/240 (52.5%)  OR: 1.53 (0.86 – 2.77)  p=0.1255 | Total score:  32/42  Selection bias:  8/14  Measurement bias:  12/14 |
| Ayub  2009 | Pakistan | 650 women | Cross sectional survey (healthcare survey of women attending primary care clinics in Lahore)  Domestic violence assessed during interview using Women Experience with Battering (WEB) and Conflict Tactics Scale (CTS) questionnaires  ICD-10 psychiatric disorders assessed during diagnostic interview using Mini International Neuropsychiatric Interview | Social phobia  Frequency of social phobic disorder (women): n= 14/650 (2.2%)  Frequency of other disorders (women): n= 396/650 (60.9%)  Frequency of no disorder (women): n= 240/650 (36.9%) | Lifetime physical and psychological intimate partner violence | *Any violence*  With phobic disorder:  n=13/14 (92.9%)  Without any disorder: n=168/240 (70%)  OR: 5.57 (0.80 – 239.97)  p=0.0662  *Physical violence*  With phobic disorder:  n=9/14 (64.3%)  Without any disorder: n=106/240 (44.2%)  OR: 2.28 (0.66 – 8.88)  p=0.1415  *Psychological violence*  With phobic disorder:  n=11/14 (78.6%)  Without any disorder: n=126/240 (52.5%)  OR: 3.32 (0.84 – 18.90)  p=0.0571 | Total score:  32/42  Selection bias:  8/14  Measurement bias:  12/14 |
| Grant  2011 | USA | 34,653 male and female sample:  n= 14,564 men  n= 20,089 women  (Prevalence data are weighted to account for survey design, oversampling and non-response) | Analysis of data from single wave of cohort study (wave II National Epidemiologic survey on alcohol and related conditions)  Domestic violence assessed during interview using DSM-IV PTSD questions  DSM-IV psychiatric disorders assessed during diagnostic interview using AUDADIS-IV | Agoraphobia  Frequency of agoraphobia (men): n=  Frequency of other disorder (men): n=  Frequency of no disorder (men): n=  Frequency of agoraphobia (women): n=  Frequency of other disorder (women): n=  Frequency of no disorder (women): n= | Lifetime physical domestic violence by an intimate partner | *Men*  With agoraphobia: 2.0% (SE 1.65)  Without any disorder:  0.8% (SE 0.11)  OR: 4.11 (0.10-25.45)  P=0.1343  *Women*  With agoraphobia: 20.7% (SE 6.07)  Without any disorder:  4.3% (SE 0.26)  OR: 5.67 (2.81-10.68)  p=0.0000 | Total score:  37/42  Selection bias:  13/14  Measurement bias:  13/14 |
| Grant  2011 | USA | 34,653 male and female sample:  n= 14,564 men  n= 20,089 women    (Prevalence data are weighted to account for survey design, oversampling and non-response) | Analysis of data from single wave of cohort study ( wave II National Epidemiologic survey on alcohol and related conditions)  Domestic violence assessed during interview using DSM-IV PTSD questions  DSM-IV psychiatric disorders assessed during diagnostic interview using AUDADIS-IV | Social or specific phobia  Frequency of social or specific phobia (men): n=2,060  Frequency of other disorder (men): n=3,935  Frequency of no disorder (men): n=8,569  Frequency of social or specific phobia (women): n=4,692  Frequency of other disorder (women): n=5,699  Frequency of no disorder (women): n=9,698 | Lifetime physical domestic violence by an intimate partner | *Men:*  With phobias: 3.8% (SE 0.50)  Without any disorder:  0.8% (SE 0.11)  OR: 4.85 (3.45-6.83)  p=0.0000  *Women:*  With phobias: 15.5% (SE 0.64)  Without any disorder:  4.3% (SE 0.26)  OR: 4.08 (3.59-4.64)  p=0.0000 | Total score:  37/42  Selection bias:  13/14  Measurement bias:  13/14 |
| Roberts 1998 | Australia | 335 women  (data on DV and MD available for 333) | Cross sectional survey (healthcare survey of 335 women attending emergency department in Brisbane)  Domestic violence assessed during interview using questions from Composite Abuse Scale (CAS)  DSM-III psychiatric disorders assessed during diagnostic interview using CIDI | Phobia  Frequency of lifetime phobia (women): n=88/333 (26.4%)  Frequency of no disorder (women): data not available | Past year physical, psychological, sexual domestic violence by an intimate partner | With phobias:  n=63/88 (71.6%) | Total score:  30/42  Selection bias:  6/14  Measurement bias:  12/14 |
| Roberts 1998 | Australia | 335 women  (data on DV and MD available for 333) | Cross sectional survey (healthcare survey of 335 women attending emergency department in Brisbane)  Domestic violence assessed during interview using questions from Composite Abuse Scale (CAS)  DSM-III psychiatric disorders assessed during diagnostic interview using CIDI | Somatisation  Frequency of lifetime somatisation (women): n=7/333 (2.1%)  Frequency of no disorder (women): data not available | Past year physical, psychological, sexual domestic violence by an intimate partner | With somatisation:  n=6/7 (85.7%) | Total score:  30/42  Selection bias:  6/14  Measurement bias:  12/14 |
| **Posttraumatic stress disorder (PTSD)** | | | | | | | |
| Ayub  2009 | Pakistan | 650 women | Cross sectional survey (healthcare survey of women attending primary care clinics in Lahore)  Domestic violence assessed during interview using Women Experience with Battering (WEB) and Conflict Tactics Scale (CTS) questionnaires  ICD-10 psychiatric disorders assessed during diagnostic interview using Mini International Neuropsychiatric Interview | Posttraumatic stress disorder (PTSD)  Frequency of PTSD (women): n=76/650 (11.7%)  Frequency of other disorders (women): n= 334/650 (51.4%)  Frequency of no disorder (women): n= 240/650 (36.9%) | Lifetime physical and psychological intimate partner violence | *Any violence*  With PTSD:  n=68/76 (89.5%)  Without any disorder: n=168/240 (70%)  OR: 3.64 (1.63 – 9.20)  p=0.0007  *Physical violence*  With PTSD:  n=47/76 (61.8%)  Without any disorder: n=106/240 (44.2%)  OR: 2.05 (1.17 – 3.61)  p=0.0072  *Psychological violence*  With PTSD:  n=61/76 (80.3%)  Without any disorder: n=126/240 (52.5%)  OR: 3.68 (1.93 – 7.34)  P=0.0000 | Total score:  32/42  Selection bias:  8/14  Measurement bias:  12/14 |
| Baker 2005 | Mexico | 2,509 male and female sample:  n= 907 men  n=1,602 women  No men reported domestic violence victimisation. | Cross sectional survey (representative community sample of 4 cities in Mexico)  Domestic violence assessed during interview using the PTSD section of CIDI, with additional questions added by author to determine age of abuse and perpetrator  DSM-IV psychiatric disorder assessed during diagnostic interview using CIDI | Posttraumatic stress disorder (PTSD)    Frequency of PTSD (women): n=80/1,602 (5.0%)  Frequency of no disorder (women): n=1,522/1,602 (95.0%) | Lifetime physical and sexual domestic violence by an intimate partner | *Women*  With PTSD  n=36/80 (45%)  Without PTSD  n=82/1522 (5.4%)  OR: 14.4 (8.46 – 24.16)  p=0.0000 | Total score:  33/42  Selection bias:  9/14  Measurement bias:  11/14 |
| Cascardi 1995 | USA | 96 women:  n=48 cases (recruited from a treatment study for serious marital conflict)  controls n=48 (recruited maritally discordant non-abused (n=23) and martially satisfied women from local newspapers (n=25)) | Case control study (community sample, cases recruited from a treatment study for serious marital conflict, controls (maritally discordant non-abused and martially satisfied women) recruited from local newspapers)  Domestic violence assessed during interview using Conflict Tactics Scale (CTS2), Psychological Maltreatment of Women Scale, Spouse-Specific Fear Measure  DSM-III psychiatric disorder assessed during diagnostic interview using SCID | Current posttraumatic stress disorder (PTSD)  Frequency of current PTSD (women): n=19/96 (19.8%).  Frequency of no disorder (women): data not available | Past year physical, psychological domestic violence by an intimate partner | With current PTSD:  n=16/19 (84.2%) | Total score:  21/42  Selection bias:  4/14  Measurement bias:  8/14 |
| Cerulli 2011 | USA | 188 women | Cross-sectional (healthcare survey in a paediatric primary care clinic)  Domestic violence assessed during interview using questions adapted from a previous study  DSM-IV psychiatric disorder assessed during diagnostic interview using SCID | Posttraumatic stress disorder (PTSD)  Frequency of current PTSD (women) n= 17/188 (9.0%)  Frequency of other disorders (women): n= 110 /188 (58.5%)  Frequency of no disorder (women): n= 61/188 (32.4%) | Lifetime physical, psychological and sexual domestic violence by an intimate partner | With PTSD:  n=7/17 (41.1%)  Without any disorder:  n=8/61 (13.1%)  OR: 4.64 (1.13 – 18.36)  p=0.0094 | Total score:  26/42  Selection bias:  6/14  Measurement bias:  10/14 |
| Contreras-Pezzotti 2011 | Colombia | 394 women:  n=132 cases  n=262 controls  (both cases and controls recruited from medical-legal primary care clinic) | Case control study (healthcare survey)    Domestic violence assessed during interview using questions developed by authors  DSM-IV psychiatric disorder assessed during diagnostic interview using SCID | Posttraumatic stress disorder (PTSD)  Frequency of PTSD (women): n= 132/394 (33.5%)  Frequency of no disorder (women): n= 262/394 (66.5%) | Lifetime violence (any type) by an intimate partner | With PTSD:  n=76/132 (57.5%)  Without any disorder:  n=85/262 (32.4%)  OR:  2.83 (1.79-4.45)  p=0.0000 | Total score:  24/42  Selection bias:  6/14  Measurement bias:  10/14 |
| Dennis 2009 | USA | 148 women  n=96 cases  n=52 controls  (both cases and controls recruited from a veteran hospital and a community hospital to obtain mixed sample of veterans and non-veterans) | Case control study (veteran and community healthcare survey)    Domestic violence assessed during interview using the Traumatic Life Events Questionnaire  DSM-IV psychiatric disorder assessed during diagnostic interview using CAPS | Posttraumatic stress disorder (PTSD)  Frequency of PTSD (women): n=72/148 (48.6%)  Frequency of other disorders (women): 24/148 (16.2%)  Frequency of no disorder (women): n=52/148 (35.1%) | Lifetime physical intimate partner violence | With PTSD:  n=42/72 (58.3%)  Without any disorder:  n=11/52 (21.2%)  OR: 5.22 (2.27 – 13.10)  p=0.0000 | Total score:  21/42  Selection bias:  3/14  Measurement bias:  11/14 |
| Duran 2009 | USA | 234 women | Cross-sectional survey (outpatient and urgent care clinic of Indian Health Service hospital in Albuquerque)  Domestic violence assessed during interview using Conflict Tactics Scale (CTS2)  DSM-IV psychiatric disorders assessed during diagnostic interview using CIDI | Posttraumatic stress disorder (PTSD)  Frequency of PTSD (women): n=34/234 (14.5%)  Frequency of other disorders (women): n= 110/234 (47.0%)  Frequency of no disorder (women): n=90/234 (38.5%) | Past year physical and sexual domestic violence by an intimate partner | With PTSD:  n=32/34 (94.1%)  Without any disorder:  n=60/90 (66.7%)  OR: 8.0 (1.80 – 72.52)  p=0.0018 | Total score:  27/42  Selection bias:  4/14  Measurement bias:  12/14 |
| Iverson  2011 | USA | 150 women | Randomised controlled trial (secondary analysis of women participating in RCT of cognitive processing therapy)  Domestic violence assessed during interview using questions adapted from the Conflict Tactics Scale (CTS)  DSM-IV psychiatric disorders assessed during diagnostic interview using CAPS. Diagnosis of PTSD was a criterion for inclusion in the trial. | Posttraumatic stress disorder (PTSD)  Frequency of PTSD (women): n=150/150 (100.0%)  Frequency of no disorder (women): n/a | Lifetime and past year physical violence by an intimate partner | Lifetime violence  With PTSD:  n=91/150 (60.7%)  *Past year violence*  With PTSD:  n=24 /150(16.0%) | Total score:  29/42  Selection bias:  7/14  Measurement bias:  11/14 |
| Kaminer 2008 | South Africa | 4,351 male and female sample:  n=1801 men  n=2550 women | Cross sectional survey (community national probability sample of 4,351 residents in South Africa )  Domestic violence assessed during interview using questions from CIDI PTSD module  DSM-IV psychiatric disorders assessed during diagnostic interview using CIDI | Posttraumatic stress disorder (PTSD):  Frequency of PTSD (women): data not available.  Frequency of no disorder (women): data not available | Lifetime physical domestic violence by an intimate partner | *Men*  OR:5.3 (0.85-32.57)  *Women*  OR:3.2 (1.48-7.10) | Total score:  33/42  Selection bias:  12/14  Measurement bias:  10/14 |
| Najavits 2004 | USA | 58 women | Intervention study (sample of 58 women participating in a psychotherapy outcome study )  Domestic violence assessed by a self-report questionnaire the Conflict Tactics Scale (CTS2)  DSM-IV psychiatric disorders assessed during diagnostic interview using SCID | Posttraumatic stress disorder (PTSD)  Frequency of PTSD (women): n=58/58(100.0%)  Frequency of no disorder (women): n/a | Past year physical, psychological and sexual intimate partner violence | *Physical violence*  With PTSD:  n=29/58 (50%)  *Psychological violence*  With PTSD:  n=50/58 (86.2%)  *Sexual violence*  With PTSD:  n=26/58 (44.8%) | Total score:  19/42  Selection bias:  2/14  Measurement bias:  9/14 |
| Grant 2011 | USA | 34,653 male and female sample:  n= 14,564 men  n= 20,089 women    (Prevalence data are weighted to account for survey design, oversampling and non-response) | Analysis of data from single wave of cohort study ( wave II National Epidemiologic survey on alcohol and related conditions)  Domestic violence assessed during interview using DSM-IV PTSD questions  DSM-IV psychiatric disorders assessed during diagnostic interview using AUDADIS-IV | Posttraumatic stress disorder (PTSD)  Frequency of PTSD (men): n=674  Frequency of any disorder (men): n=5,321  Frequency of no disorder (men): n=8,569  Frequency of PTSD (women): n=1,789  Frequency of any disorder (women): n=8,602  Frequency of no disorder (women): n=9,698 | Lifetime physical domestic violence by an intimate partner | *Men*  With PTSD: 7.3% (SE 1.27)  Without any disorder:  0.8% (SE 0.11)  OR: 9.66 (6.49-14.26)  p=0.0000  *Women*  With PTSD: 29.4% (SE 1.27)  Without any disorder:  4.3% (SE 0.26)  OR: 9.27 (8.03-10.70)  p=0.0000 | Total score:  37/42  Selection bias:  13/14  Measurement bias:  13/14 |
| Schumacher  2010 | USA | 445 male and female sample:  n= 194 men  n= 251 women  (Gender disaggregated domestic violence data not available). | Cross sectional survey (community sample of residents affected by Hurricane Katrina residing in 23 counties of Mississippi)  Domestic violence assessed during interview using questions developed by the authors  DSM-IV psychiatric disorders assessed during diagnostic interview using CIDI | Posttraumatic stress disorder (PTSD) following Hurricane Katrina:  Frequency of PTSD (men): n=25/194 (14.3%, weighted prevalence)  Frequency of no disorder (men): n=169/194 (85.7%, weighted prevalence)  Frequency of PTSD (women): n=37/251 (14.6%, weighted prevalence)  Frequency of no disorder (women): n=217/251 (85.4%, weighted prevalence) | Physical and psychological domestic violence by an intimate partner occurring in the six months prior to or following Hurricane Katrina. | *Men and Women:*  *Physical violence*  OR: 2.58 (1.42-4.68)  p=0.002  *Psychological violence*  OR: 3.27 (1.71-6.27)  p<0.001 | Total score:  33/42  Selection bias:  11/40  Measurement bias:  11/40 |
| Tolman 2001 | USA | 753 women | Analysis of single wave of cohort study (wave 1 of Women’s Employment Study of female welfare recipients)  Domestic violence assessed during interview using questions from Conflict Tactics Scale (CTS)  DSM-IV psychiatric disorders assessed during diagnostic interview using UM-CIDI. Short form scales were used to assess depression and GAD, PTSD was assessed using the full scale. | Posttraumatic stress disorder (PTSD)  Frequency of lifetime PTSD (women): n= 222/753 (29.5%)  Frequency of other disorders (women): n=35/753 (4.6%)  Frequency of no disorders (women): n=496/753 (65.9%) | Past year and lifetime severe physical domestic violence by an intimate partner | *Lifetime violence*  *Lifetime PTSD*  With PTSD:  n=171/222 (77.0%)  Without any disorder:  202/496 (40.7%)  OR: 4.88 (3.36 – 7.14)  p=0.0000  *Past year violence*  *Lifetime PTSD*  With PTSD:  n=60/222 (27.0%)  Without any disorder:  n=46/496 (9.3%)  OR: 3.62 (2.32 – 5.67)  p=0.0000 | Total score:  29/42  Selection bias:  9/14  Measurement bias:  12/14 |
| Yasan 2009 | Turkey | 708 male and female sample:  n=310 men  n= 398 women  No men reported domestic violence victimisation. | Cross sectional survey (community survey of residents living in provincial centre of Diyarbakir)  Domestic violence assessed during interviews as a traumatic event which was consistent with Criterion A of the CAPS  DSM-IV psychiatric disorders assessed during diagnostic interview using CAPS | Posttraumatic stress disorder (PTSD)  Frequency of current PTSD (women): n=55/398 (13.9%)  Frequency of no disorder (women): n=343/398 (86.1%) | Lifetime physical and sexual domestic violence (perpetrator not specified). | *Women*  With PTSD:  n=19/55 (34.5%)  Without any disorder:  n=10/343 (2.9%)    OR: 17.58 (7.05 – 45.19)  p=0.0000 | Total score:  26/42  Selection bias:  6/14  Measurement bias:  13/14 |
| **Obsessive-compulsive disorder (OCD)** | | | | | | | |
| Ayub  2009 | Pakistan | 650 women | Cross sectional survey (healthcare survey of women attending primary care clinics in Lahore)  Domestic violence assessed during interview using Women Experience with Battering (WEB) and Conflict Tactics Scale (CTS) questionnaires  ICD-10 psychiatric disorders assessed during diagnostic interview using Mini International Neuropsychiatric Interview | Obsessive compulsive disorder (OCD)  Frequency of OCD (women):  n= 48/650 (7.4%)  Frequency of other disorders (women): n=362 /650 (55.7%)  Frequency of no disorder (women): n= 240/650 (36.9%) | Lifetime physical and psychological intimate partner violence | *Any violence*  With OCD:  n=45/48 (93.8%)  Without any disorder: n=168/240 (70%)  OR: 6.43 (1.95 – 33.23)  p=0.0006  *Physical violence*  With OCD:  n=20/48 (41.7%)  Without any disorder: n=106/240 (44.2%)  OR: 0.90 (0.46 – 1.77)  p=0.7499  *Psychological violence*  With OCD:  n=43/48 (89.6%)  Without any disorder: n=126/240 (52.5%)  OR: 7.78 (2.93 – 25.89)  p=0.0000 | Total score:  32/42  Selection bias:  8/14  Measurement bias:  12/14 |
| Cerulli 2011 | USA | 188 women | Cross-sectional (healthcare survey in a paediatric primary care clinic)  Domestic violence assessed during interview using questions adapted from a previous study  DSM-IV psychiatric disorder assessed during diagnostic interview using SCID | Obsessive compulsive disorder (OCD)  Frequency of current OCD (women): n=11/188 (5.9%)  Frequency of other disorders (women): n= 116/188 (61.7%)  Frequency of no disorder (women): n= 61/188 (32.4%) | Lifetime physical, sexual and psychological partner violence | With OCD:  n=4/11 (36.3%)  Without any disorder:  n=8/61 (13.1%)  OR: 3.79 (0.65 – 19.09)  p=0.0569 | Total score:  26/42  Selection bias:  6/14  Measurement bias:  10/14 |
| **Eating Disorders** | | | | | | | |
| Danielson 1998 | New Zealand | 922 male and female sample:  N=461 women  N=480 men  (victimisation not measured in men | Analysis of data from single wave of cohort study (Dunedin birth cohort, using data collected on psychiatric disorders and violence when respondents aged 21 years)  Domestic violence assessed during interview using Conflict Tactics Scale (CTS)  DSM-III psychiatric disorder assessed during diagnostic interview using DIS | Eating disorder  Frequency of eating disorder (women): n=11/461 (2.3%)  Frequency of other disorders (women): n= 186/461 (40.3%)  Frequency of no disorder (women): n=264/461 (57.3%) | Past year physical domestic violence by an intimate partner | With eating disorder:  n=7/11 (63.6%)  Without any disorder:  n=51/264 (19.3%)  OR: 7.31 (1.76 – 35.10)  p=0.0004 | Total score:  33/42  Selection bias:  11/14  Measurement bias:  12/14 |
| **Personality disorders** | | | | | | | |
| Danielson 1998 | New Zealand | 922 male and female sample  n=461 women  n=480 men  (victimisation not measured in men) | Analysis of data from single wave of cohort study (Dunedin birth cohort, using data collected on psychiatric disorders and violence when respondents aged 21 years)  Domestic violence assessed during interview using Conflict Tactics Scale (CTS)  DSM-III psychiatric disorder assessed during diagnostic interview using DIS | Antisocial personality disorder (ASPD):  Frequency of ASPD (women): n=3/461 (0.6%)  Frequency of other disorders (women): 194/461 (42.1%)  Frequency of no disorder (women): n=264/461 (57.3%) | Past year physical domestic violence by an intimate partner | With ASPD:  n=3/3 (100%)  Without any disorder:  n=51/264 (19.3%)  OR: (n/a) | Total score:  33/42  Selection bias:  11/14  Measurement bias:  12/14 |
| Grant  2011 | USA | 34,653 male and female sample  n= 14,564 men  n= 20,089 women    (Prevalence data are weighted to account for survey design, oversampling and non-response) | Analysis of data from single wave of cohort study (wave II National Epidemiologic survey on alcohol and related conditions)  Domestic violence assessed during interview using DSM-IV PTSD questions  DSM-IV psychiatric disorders assessed during diagnostic interview using AUDADIS-IV | Any personality disorder  Frequency of personality disorder (men):  n=3,498  Frequency of other disorder (men): n=2,497  Frequency of no disorder (men): n=8,659  Frequency of personality disorder (women):  n=4,285  Frequency of other disorder (women): n=6,106  Frequency of no disorder (women): n=9,698 | Lifetime physical domestic violence by an intimate partner | *Men*  With personality disorder: 5.4% (SE 0.50)  Without any disorder:  0.8% (SE 0.11)  OR:7.04 (5.30-9.43)  p=0.000  *Women*  With personality disorder: 21.4% (SE 0.86)  Without any disorder:  4.3% (SE 0.26)  OR: 6.06 (5.35-6.86)  p=0.0000 | Total score:  37/42  Selection bias:  13/14  Measurement bias:  13/14 |
| Weizmann-Hennelius  2004 | Finland | 91 women:  N=61 cases  (violent offenders incarcerated or hospitalised in Finland during a 12 month period)  30 controls  (non-offender women recruited from community education or evening courses)  Data available on DV and MD for 89 women. | Case control study (cases were female violent offenders incarcerated or hospitalised during a 12 month period and controls were non-offender women recruited from community education or evening courses)  Domestic violence assessed during interview using questions developed by the authors  DSM-IV psychiatric disorders assessed during diagnostic interview using SCID | Any personality disorder:  Frequency of personality disorder (women): n=50/89 (56.2%)  Frequency of no disorder (women): n=39/89 (43.8%) | Lifetime physical, sexual and psychological violence by an intimate partner; past year physical and sexual violence by an intimate partner. | *Any lifetime violence*  With personality disorder:  n=38/50 (76.0%)  Without any disorder:  n=23/39 (76.0%)  2.20 (0.81 – 6.07)  p=0.0861  *Lifetime physical violence*  With personality disorder:  n=33/50 (66.0%)  Without any disorder:  n=12 /39 (30.8%)  OR: 4.37 (1.63 – 11.87)  p=0.0010  *Lifetime sexual violence*  With personality disorder:  n=9/50 (18%)  Without any disorder:  n=3/39 (7.7%)  OR: 2.63 (0.59 – 16.11)  p=0.1578  *Lifetime psychological violence*  With personality disorder:  n=26 /50(52.0%)  Without any disorder:  n=13 /39 (33.3%)  OR: 2.17 (0.84 – 5.67)  p=0.0782  *Any past year violence*  With personality disorder:  n=19/50 (38.0%)  Without any disorder:  n=9/39 (23.1%)  OR: 2.04 (0.73 – 5.95)  p=0.1325  *Past year physical violence*  With personality disorder:  n=18/50 (36.0%)  Without any disorder:  n=7/39 (17.9%)  OR: 2.57 (0.87 – 8.25)  p=0.0601  *Past year psychological violence*  With personality disorder:  n=10/50 (20.0%)  Without any disorder:  n=8 /39 (20.5%)  OR: 0.97 (0.30 – 3.19)  p=0.9523 | Total score:  28/42  Selection bias:  5/14  Measurement bias:  10/14 |
| Zanarini 1999 | USA | 290 mixed:  N=290 cases  (men and women diagnosed with borderline personality disorder)  N=72 controls  (men and women with axis II disorders) | Case control study (all participants recruited from inpatient mental health centre)  Domestic violence assessed during interview using Abuse History Interview  DSM-III-R and DIB-R psychiatric disorders assessed during diagnostic interview using SCID | Borderline personality disorder (BPD)  Frequency of BPD (men and women): n=290/362 (80.1%)  Frequency of no disorder (men and women): n/a – psychiatric sample. | Lifetime physical partner violence | With BPD  96/290 (33.1%) | Total score:  31/42  Selection bias:  7/14  Measurement bias:  11/14 |
| **Other disorders** | | | | | | | |
| Ayub  2009 | Pakistan | 650 women | Cross sectional survey (healthcare survey of women attending primary care clinics in Lahore)  Domestic violence assessed during interview using Women Experience with Battering (WEB) and Conflict Tactics Scale (CTS) questionnaires  ICD-10 psychiatric disorders assessed during diagnostic interview using Mini International Neuropsychiatric Interview | Dysthymia  Frequency of dysthymia (women): n=37/650 (5.7%)  Frequency of other disorders (women): n= 373/650 (57.4%)  Frequency of no disorder (women): n= 240/650 (36.9%) | Lifetime physical and psychological intimate partner violence | *Any violence*  With dysthymic disorder:  n=36/37 (97.3%)  Without any disorder: n=168/240 (70%)  OR: 15.43 (2.48 – 634.54)  P=0.0005  *Physical violence*  With dysthymic disorder:  n= 20/37 (54.1%)  Without any disorder: n=106/240 (44.2%)  OR: 1.49 (0.70 – 3.18)  P=0.2609  *Psychological violence*  With dysthymic disorder:  n=34/37 (91.9%)  Without any disorder: n=126/240 (52.5%)  OR: 10.25 (3.08 – 53.24)  p=0.0000 | Total score:  32/42  Selection bias:  8/14  Measurement bias:  12/14 |
| Cascardi 1995 | USA | 96 women:  n=48 cases (recruited from a treatment study for serious marital conflict)  controls n=48 (recruited maritally discordant non-abused (n=23) and martially satisfied women from local newspapers (n=25)) | Case control  (community sample)  Domestic violence assessed during interview using Conflict Tactics Scale (CTS2), Psychological Maltreatment of Women Scale, Spouse-Specific Fear Measure  DSM-III psychiatric disorder assessed during diagnostic interview using SCID | Dysthymia  Frequency of dysthymia (women): n= 9/96 (9.4%)  Frequency of no disorder (women): data not available | Past year physical, psychological domestic violence by an intimate partner | With dysthymic disorder:  n=6/9 (66.7%) | Total score:  21/42  Selection bias:  4/14  Measurement bias:  8/14 |
| Fisher 2010 | Vietnam | 364 women | Cross-sectional survey (community sample of women who were >7 months pregnant or 4.8 weeks postpartum and registered with commune health centres in two Vietnamese provinces)  Domestic violence assessed during interview using questions developed by authors. Psychological abuse assessed by inquiring about fear of partner  DSM-IV psychiatric disorders assessed during diagnostic interview using SCID | Dysthymia  Frequency of dysthymia (women): n=6/364 (1.6%)  Frequency of other disorders (women): n= 103/364 (28.3%)  Frequency of no disorder (women): 255/364 (70.1%) | Past year physical and psychological violence by an intimate partner. | *Any violence*  With dysthymic disorder:  n=1/6 (16.7%)  Without any disorder:  n=32/255 (13.7%)  OR: 1.39 (0.03 – 13.00)  p=0.7642  *Physical violence*  With dysthymic disorder:  n=0/6 (0.0)  Without any disorder:  n=5/255 (2.0%)  OR: n/a  *Psychological violence*  With dysthymic disorder:  n=1/6 (16.7%)  Without any disorder:  n=35/255 (12.5%)  OR: 1.26 (0.26 – 11.68)  p=0.8364 | Total score:  33/42  Selection bias:  12/14  Measurement bias:  11/14 |
| Grant  2011 | USA | 34,653 male and female sample  n= 14,564 men  n= 20,089 women  (data weighted to account for survey design, oversampling and non-response) | Analysis of data from single wave of cohort study (wave II National Epidemiologic survey on alcohol and related conditions)  Domestic violence assessed during interview using DSM-IV PTSD questions  DSM-IV psychiatric disorders assessed during diagnostic interview using AUDADIS-IV | Dysthymia  Frequency of dysthymia (men): n=370  Frequency of other disorders (men): 5,625  Frequency of no disorder (men): n=8,569  Frequency of dysthymia (women): n=938  Frequency of other disorders (women): 9,453  Frequency of no disorder (women): n=9,698 | Lifetime physical domestic violence by an intimate partner | *Men*:  With dysthymic disorder: 3.9% (SE 1.04)  Without any disorder:  0.8% (SE 0.11)  OR: 4.84 (2.49-8.79)  p=0.0000  *Women:*  With dysthymic disorder: 20.0% (SE 1.48)  Without any disorder:  4.3% (SE 0.26)  OR: 5.58 (4.60-6.76)  p=0.0000 | Total score:  37/42  Selection bias:  13/14  Measurement bias:  13/14 |
| Roberts 1998 | Australia | 335 women  (analysis only available for 333 women) | Cross sectional survey (healthcare survey of 335 women attending emergency department in Brisbane).  Domestic violence assessed during interview using questions from Composite Abuse Scale (CAS)  DSM-III psychiatric disorders assessed during diagnostic interview using CIDI | Lifetime dysthymia  Frequency of dysthymia (women): n=39/333 (11.7%).  Frequency of no disorder (women): data not available | Past year physical, psychological, sexual domestic violence by an intimate partner | With dysthymic disorder:  n=29 (74.4%) | Total score:  30/42  Selection bias:  6/14  Measurement bias:  12/14 |
