# Supplementary material for: Experiences of Domestic Violence and Mental Disorders: A Systematic Review and Meta-Analysis
Source: PLoS One. 2012 Dec 26;7(12):e51740. doi: 10.1371/journal.pone.0051740 (PMC3530507; doi:10.1371/journal.pone.0051740)
Supplement: Table S2 — Critical Appraisal Checklist for Included Studies. (DOC) [file pone.0051740.s006.doc]

Quality Appraisal Form

*Please complete part 1 for all study designs and complete the relevant sections for part 2, specific to study design.*

Score the answer to each question by ticking 0, 1 or 2:

0 – study does not meet criteria/answer question

1 – Study partially meets criteria/gives a partially satisfactory answer to the question

2 – Study fully meets criteria/gives a fully satisfactory answer to the question

| **Part 1** | | | | | | | | | |
| --- | --- | --- | --- | --- | --- | --- | --- | --- | --- |
| **Screening questions** | | | | | | **Score** | | | |
|  | Question | | Comments | | | 0 | | 1 | 2 |
| 1 | Did the study ask a clearly focused question?  *– Is the hypothesis/aim/objective of the study clearly described?*  *-Is the study question focused in terms of the outcomes considered?* | |  | | |  | |  |  |
| 2 | Is the study design appropriate for the research question? | |  | | |  | |  |  |
| 3 | Was a validated tool used to assess mental disorder?   - - *Validated diagnostic instrument used (i.e., in diagnostic interview or case file assessment) =2*   - *Validated screening instrument used (i.e., in screening interview or case file assessment) =1* | |  | | |  | |  |  |
| **Continue only if score on each of questions 1 and 2 is one or more** | | | | | | | | | |
| **Detailed questions** | | | | | | | | | |
| **Measurement of risk of selection bias** | | | | | | | | | |
| 4a | | Is the sampling method appropriate for the research question?  *Consider:*  *-The sampling method used (i.e. random selection of subjects)*  *- If applicable, is there appropriate selection of controls?* | |  |  | |  | |  |
| 4b | | Are subjects appropriately defined?  *Consider:*  *- Inclusion/ exclusion criteria specified*  *- Inclusion/exclusion criteria appropriate* | |  |  | |  | |  |
| 4c | | Is the sample size appropriate?  *Consider:*  *- Is the sample size justified?*  *- Were a sufficient number of cases selected?*  *- If applicable, were a sufficient number of controls selected?* | |  |  | |  | |  |
| 4d | | Is the study sample representative of the population of interest?  *-Do the authors assess the representativeness of the study sample?* | |  |  | |  | |  |
| 4e | | Does the level of non-participation risk introducing bias?  *Consider:*  *-Are key demographic characteristics of non-participants reported and compared against participants?*  *-Does the study report on the impact of non-participation?*  *-If applicable, rates of attrition reported* | |  |  | |  | |  |
| 5 | | Is the study setting appropriate to the aims of the research? (e.g. setting, location, relevant dates) | |  |  | |  | |  |
| 6 | | Is the method of data collection appropriate for the aims of the research? | |  |  | |  | |  |
| **Measurement of risk of reporting bias** | | | | | | | | | |
| 7 | | Are suitable/standard criteria used for measurement of domestic violence?  *Consider:*  *-Criteria of domestic violence was clearly defined*  *-Potential for bias of measurement*  *-If measures piloted*  *- Standardised/pre-validated measures (score 2 points)*  *- Researchers developed their own measure (score 1 point)*  *- No details of measurement were provided (score 0 point)* | |  |  | |  | |  |
| 8 | | Are known confounders accounted for by study design?  *- Was consideration of confounding factors accounted for in study design?* | |  |  | |  | |  |
| 9 | | Are known confounders accounted for in the analyses? | |  |  | |  | |  |
| 10 | | Are the statistical tests used to assess the main outcomes appropriate?  *-Was there adequate adjustment for confounding in the analyses?*  *- Do the analyses adjust for different lengths of follow-up (if applicable)?* | |  |  | |  | |  |
| 11a | | Are the estimates reported with confidence intervals and in detail by sub-group (if appropriate)?  *- Were the findings reported clearly?* | |  |  | |  | |  |
| 11b | | Are statistically non-significant results presented? | |  |  | |  | |  |
| 11c | | Are data for relevant variables complete? | |  |  | |  | |  |
| 12 | | Was the conduct of the fieldwork appropriate to the study setting?  *-Was the allocation of the interviewer/interpreter sensitive to the background of the participant?*  *-Were fieldworkers trained and supported to work with people who have experienced domestic violence?* | |  |  | |  | |  |
| 13 | | Were ethical considerations appropriately considered?  -*Did researchers obtain informed consent from all participants?*  *- Did researchers take adequate precautions to safeguard participant anonymity and confidentiality?*  *-Did fieldworkers offer information about domestic violence support and referral options to all participants?*  -*Were fieldworkers appropriately trained to deal with participant distress?* | |  |  | |  | |  |
| 14 | | Do the findings support the conclusions? | |  |  | |  | |  |
| 15 | | Are the strengths and weaknesses of the research discussed? | |  |  | |  | |  |

Calculate total score of part 1 (out of a possible total of 42):
