# Supplementary figures and images for: Experiences of Domestic Violence and Mental Disorders: A Systematic Review and Meta-Analysis
Source: PLoS One. 2012 Dec 26;7(12):e51740. doi: 10.1371/journal.pone.0051740 (PMC3530507; doi:10.1371/journal.pone.0051740)

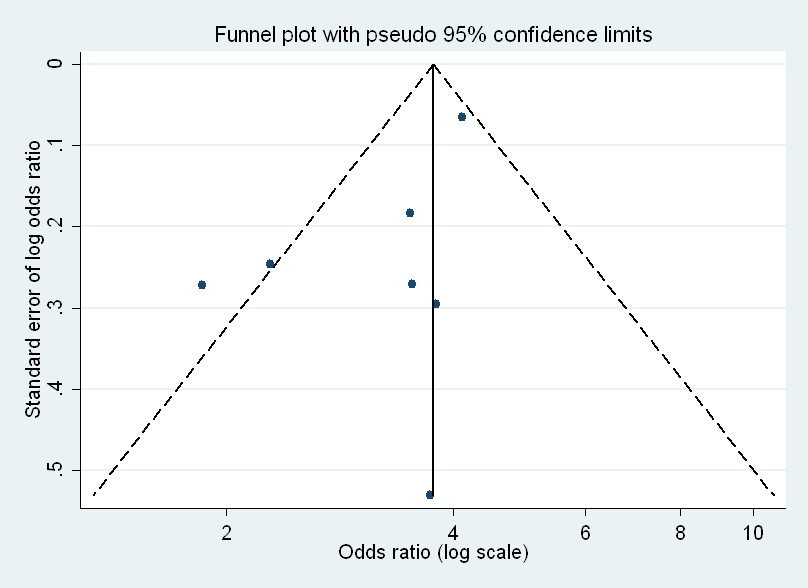

Supplement: Figure S1 — Funnel plot: Odds of lifetime domestic violence among women with depressive disorder. (TIF) [file pone.0051740.s008.tif]

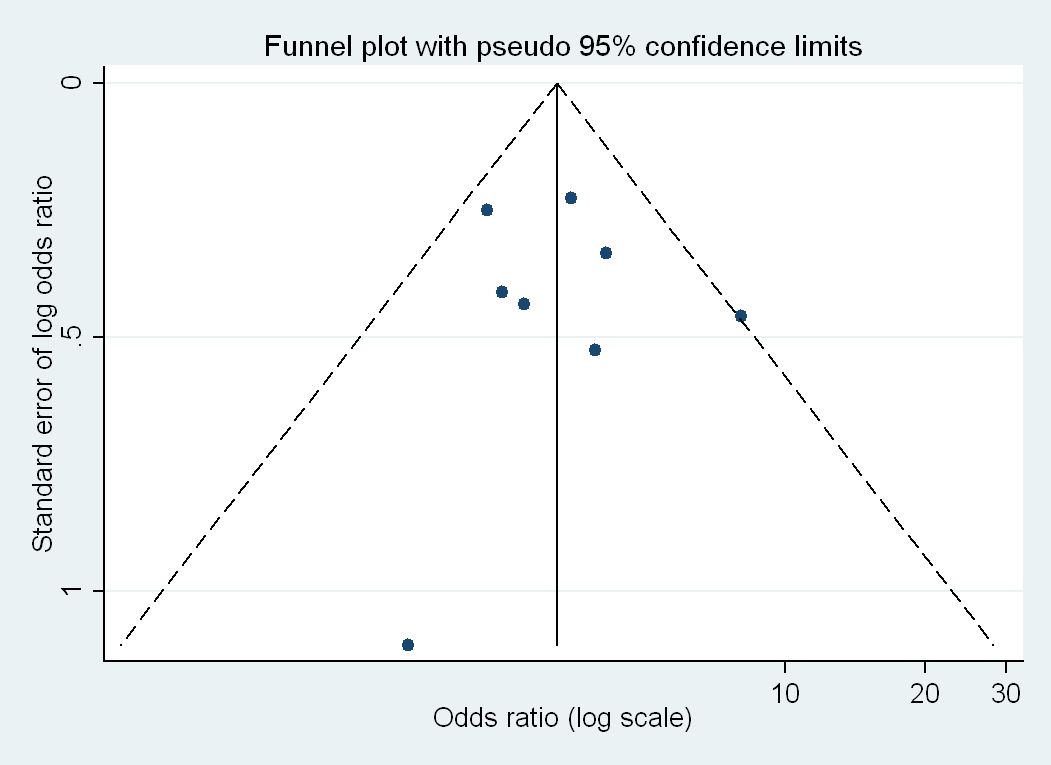

Supplement: Figure S2 — Funnel plot: Odds of past year domestic violence among women with depressive disorder. (TIF) [file pone.0051740.s009.tif]

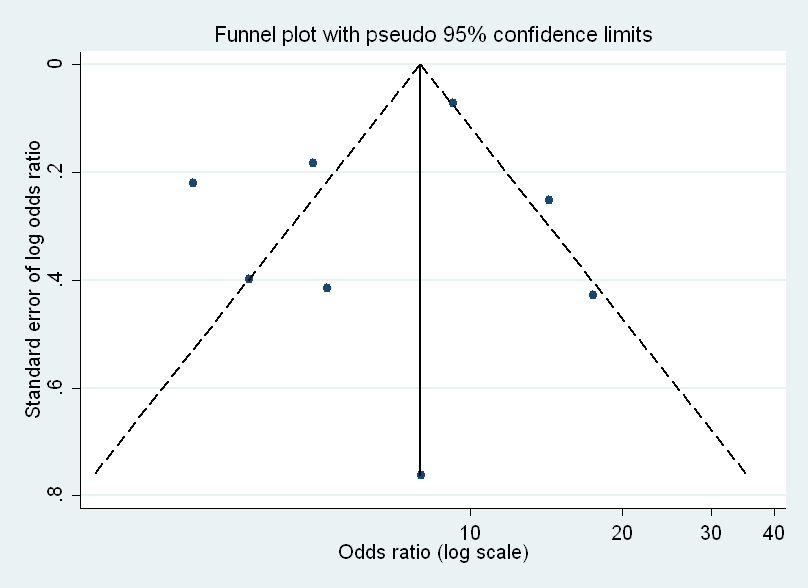

Supplement: Figure S3 — Funnel plot: Odds of lifetime domestic violence among women with post-traumatic stress disorder. (TIF) [file pone.0051740.s010.tif]
